# Supplementary material for: Patrilineal segmentary systems provide a peaceful explanation for the post-Neolithic Y-chromosome bottleneck
Source: Nat Commun. 2024 Apr 24;15:3243. doi: 10.1038/s41467-024-47618-5 (PMC11043392; doi:10.1038/s41467-024-47618-5)
Supplement: Supplementary file 1 — Supplementary Information [file 41467_2024_47618_MOESM1_ESM.pdf]

Supplementary Information

Patrilineal segmentary systems provide a peaceful explanation for the post-Neolithic Y-chromosome bottleneck

Supplementary note 1: Effect of different scenarios on global π-based male and female effective population size

When computing global (over all villages) π-based effective population sizes, scenarios without post-fission migration showed a constant male effective population size and female-to-male N_e ratio over time, except in scenarios 2d and 2h. In these latter scenarios, where there is violent intergroup competition and lineal fission, male effective population size decreased by a factor comprised between 1.1 and 1.4, 100 generations after the introduction of the kinship rules. Therefore, female-to-male N_e ratio increased over time, reaching values between 1.1 and 1.4, 100 generations after the introduction of the kinship rules (Supplementary Tables 7-10, Supplementary Fig. 7-10). Consistently, global π-based male effective population size slightly increased in scenarios 3a to 3c (where there is no violent competition between descent groups) due to exponential demographic growth, resulting in constant female-to-male N_e ratio over time (Supplementary Table 7, Supplementary Fig. 7). However, when post-fission migration was considered, the same trends were observed for the change in the global π-based male effective population size and in the global π-based female-to-male N_e as for local (within village) π-based effective population size. This can be explained by the fact that patrilineal groups can migrate between villages, making Y-chromosome diversity within villages more representative of the global Y chromosome diversity. In scenarios considering only post-fission migration (and no 2% intervillage male migration), values of the female-to-male N_e ratio ranged from 0.92 (scenario 1 and 2a) to 7.01 (scenario 2g), 100 generations after the introduction of the kinship rules (Supplementary Table 9, Supplementary Fig. 9). In the scenarios with post-fission migration and 2% intervillage male migration, the female-to-male N_e ratio ranged from 0.89 (scenario 1) to 3.28 (scenario 2g), 100 generations after the introduction of the kinship rules (Supplementary Table 10, Supplementary Fig. 10). As for local π-based effective population size estimates, considering a small percentage (2%) of intervillage male migration in the model mitigates the reduction in the global π-based male effective population size.

Supplementary note 2: Combined effect of variance in reproductive success and violence on π-based male effective population size

The combination of variance in reproductive success between groups and violent competition can have a weaker effect on male effective population size than variance alone. For example, the π-based reduction factor was 3.70 in scenario 2e, while it was 2.74 in scenario 2f, without post-fission migration and without 2% male migration. This is because violence is coded to have a greater effect on small groups, as in Zeng *et al.* [9]. This may limit the potential of small groups in expansion to replace other groups (if they have a larger reproductive success).

Supplementary tables

Supplementary Table 1: Extinction rates of patrilineal lines and groups in the literature and in the model presented in this study

**Study**	**Extinction rates per generation**	**Geographical area and time period**
Birdsell 1968 [104]	*No information on method*<br><br>**0.3**	Australian patrilineal society<br>20th century
Song *et al.* 2015 [45]	*Obtained from the average extinction rate of descent lines over 150 years within the Imperial lineage:*<br><br>- high status: **0.08**<br>- low status: **0.125**	China<br>18th and 19th centuries
Kolk *et al.* 2022 [105]	*Obtained from the average extinction rate of descent lines over 3 generations*<br><br>**0.16**	Northern Sweden<br>Between 1885 and 1899
Present study	*Average extinction rates of descent groups over all replicates and all generations for the scenario 2g without 2% intervillage male migration and without post-fission migration with different values of the parameter controlling variance in reproductive success (σ^2) between descent groups*<br><br>$\sigma^2 = 0.05$<br>(low variance)<br>**0.02**<br><br>$\sigma^2 = 0.1$<br>(intermediate variance)<br>**0.03**<br><br>$\sigma^2 = 0.2$<br>(high variance)<br>**0.05**	

Supplementary Table 2: Growth rates of patrilineal lines and groups in the literature and in the model presented in this study

**Study**	**Exponential growth rates per generation**	**Geographical area and time period**
Forde 1938 [55]	Ndai patrilineage: **0.19**<br>*computed from Table IV over 1 generation*	Southern Nigeria<br>20th century
Davis 1986 [106]	Shih Hao lineage: **2.5**<br>*computed from Table 3.1 over 10 generations*	China<br>960-1279 CE
Gabrilopoulos *et al.* 2002 [107]	Kugri patrilineage: **2.08**<br>*computed from Figure 3 over 3 generations*	Northern Ghana<br>Contemporary to the study
Moore 2006 [108]	Ui Néill lineage: **0.19**	Ireland<br>from 500 BP to present
Song *et al.* 2015 [45]	*Obtained from the average growth rates of descent lines over 150 years within the Imperial lineage:*<br><br>- high status: **0.18**<br>- low status: **-0.0077**<br><br>*and the Liaoning lineage:*<br><br>- high status: **0.12**<br>- low status: **-0.018**	China<br>18th and 19th centuries
Present study	*Average growth rates of growing descent groups over all replicates and all generations for the scenario 2g without 2% intervillage male migration and without post-fission migration with different values of the parameter controlling variance in reproductive success (σ^2) between descent groups*<br><br>σ^2 = 0.05<br>(low variance)<br>**0.13**<br><br>σ^2 = 0.1<br>(intermediate variance)<br>**0.14**<br><br>σ^2 = 0.2<br>(high variance)<br>**0.16**	

Supplementary Table 3: Comparison of parameters values with Zeng *et al.* [9]

Patrilineal kin groups are called descent groups in our study, following Fox's terminology [10], and cultural groups or kin groups in Zeng *et al.*'s study [9].

Parameter values	**Present study**	**Zeng *et al.* [9]**
Y chromosome mutation rate	2.5×10^{-8} mut/nuc/gen	2×10^{-3} mut/haplogroup/gen
mtDNA mutation rate	5.5×10^{-7} mut/nuc/gen	None
Nb of replicates	200	100
Nb of generations of patriliny	100	60
Nb of villages	5	NA
Initial nb of patrilineal groups	15 (3 per village)	100
Initial nb of individuals	300 (males and females) per village	10,000 (only males)
Initial nb of individuals per patrilineal group	100 (males and females)	100 (only males)
Reproduction	villages of increasing size; each descent group has a growth rate and transmits it with variance to the newly formed descent groups at the moment of fission.	population of constant size; at the end of each generation, cultural groups grow proportionately to their size.
Condition for fission	When the descent group reaches a fixed threshold and if more than 3 generations have passed since the last fission event.	When a cultural group goes extinct.
Fission type	If the fission is random, the splitting descent group divides randomly in two. The ratio of number of males between the newly born groups is drawn in a truncated normal law of parameter 1/2. If the fission is lineal, the splitting descent group is divided so that newly formed groups gather the most paternally related males.	The splitting cultural group divides randomly into two halves.
Extinction threshold	0 individuals	20 individuals
Variance in reproductive success between patrilineal groups	none, low, intermediate, high	none or cultural selection (i.e. the fitness varies between groups)

Supplementary Table 4: π-based and coalescent-based male effective population size reduction factor and maximum ratio of female-to-male effective population size under different scenarios without post-fission migration and with 2% intervillage male migration.

In the scenarios presented here, males migrate at a rate of 2% between villages. All other parameters are identical to those in Table 2.
* calculated by dividing the number of males in the simulation at t_0 (i.e. 750) by the mean π-based male effective population size, 100 generations after t_0.
** calculated by dividing the number of males in the simulation at t_0 (i.e. 750) by the minimum coalescent-based male effective population size.
*** The reported values were obtained from visual inspection of Karmin *et al.*' figure S4B [1].

Scenario / Study	Male effective population size reduction factor		Maximum female-to-male effective population size ratio	
	π-based *	coalescent-based **	π-based	coalescent-based
Karmin *et al.* [1] ***	unknown	Siberia: 2.4 ; Andes: 2.5<br>South-East and East Asia: 2.7<br>South and Central Asia: 2.9<br>Near East: 3.7 ; Africa: 4<br>Europe: 6.7<br>3.5 on average	unknown	17
1	0.95	0.84	0.96	1.37
2a	0.99	0.88	1.05	1.30
2b	1.12	1.05	1.15	1.79
2c	0.95	0.84	0.97	1.31
2d	1.73	1.90	1.81	3.48
2e	1.35	1.36	1.41	3.35
2f	1.28	1.33	1.30	2.70
2g	1.73	2.28	1.73	6.07
2h	2.42	3.19	2.46	7.26

Supplementary Table 5: π-based and coalescent-based male effective population size reduction factor and maximum ratio of female-to-male effective population size under different scenarios with post-fission migration and without intervillage male migration.

In the scenarios presented here, males do not migrate between villages and there is post-fission migration. All other parameters are identical to those in Table 2.
* calculated by dividing the number of males in the simulation at t_0 (i.e. 750) by the mean π-based male effective population size, 100 generations after t_0.
** calculated by dividing the number of males in the simulation at t_0 (i.e. 750) by the minimum coalescent-based male effective population size.
*** The reported values were obtained from visual inspection of Karmin *et al.*' figure S4B [1].

Scenario / Study	Male effective population size reduction factor		Maximum female-to-male effective population size ratio	
	π-based *	coalescent-based **	π-based	coalescent-based
Karmin *et al.* [1] ***	unknown	Siberia: 2.4 ; Andes: 2.5<br>South-East and East Asia: 2.7<br>South and Central Asia: 2.9<br>Near East: 3.7 ; Africa: 4<br>Europe: 6.7<br>3.5 on average	unknown	17
1	1.31	0.81	1.29	1.29
2a	0.99	0.90	1.01	1.31
2b	1.39	1.56	1.43	2.59
2c	1.06	0.85	1.08	1.30
2d	3.24	4.00	3.21	8.51
2e	2.19	4.02	2.15	9.87
2f	2.15	3.03	2.16	7.78
2g	10.39	7.70	10.72	21.44
2h	12.33	7.87	12.23	23.50

Supplementary Table 6: π-based and coalescent-based male effective population size reduction factor and maximum ratio of female-to-male effective population size under different scenarios with post-fission migration and 2% intervillage male migration.

In the scenarios presented here, males migrate at a rate of 2% between villages and there is post-fission migration. All other parameters are identical to those in Table 2.
* calculated by dividing the number of males in the simulation at t_0 (i.e. 750) by the mean π-based male effective population size, 100 generations after t_0.
** calculated by dividing the number of males in the simulation at t_0 (i.e. 750) by the minimum coalescent-based male effective population size.
*** The reported values were obtained from visual inspection of Karmin *et al.*' figure S4B [1].

Scenario / Study	Male effective population size reduction factor		Maximum female-to-male effective population size ratio	
	π-based *	coalescent-based **	π-based	coalescent-based
Karmin *et al.* [1] ***	unknown	Siberia: 2.4 ; Andes: 2.5<br>South-East and East Asia: 2.7<br>South and Central Asia: 2.9<br>Near East: 3.7 ; Africa: 4<br>Europe: 6.7<br>3.5 on average	unknown	17
1	0.95	0.84	0.96	1.37
2a	0.96	0.87	0.98	1.37
2b	1.14	1.27	1.10	2.31
2c	0.95	0.86	0.97	1.33
2d	1.87	2.62	1.82	4.98
2e	1.60	2.91	1.64	7.33
2f	1.30	2.22	1.31	4.92
2g	4.27	4.86	4.42	14.20
2h	3.78	4.90	3.68	12.82

Supplementary Table 7: Global π-based male effective population size reduction factor and maximum ratio of female-to-male effective population size under different scenarios with no post-fission migration or 2% intervillage male migration.

Scenarios settings and parameter values are the same as for the scenarios in Table 2 but the π-based effective population sizes are computed by sampling individuals in all villages.

Scenario / Study	Global π-based male effective population size reduction factor	Global π-based maximum female-to-male effective population size ratio
1	0.92	0.91
2a	0.94	0.92
2b	0.93	0.92
2c	0.90	0.90
2d	1.29	1.28
2e	1.01	1.05
2f	0.97	1.01
2g	0.93	0.96
2h	1.14	1.17
3a	0.89	0.97
3b	0.86	0.94
3c	0.86	0.96

Supplementary Table 8: Global π-based male effective population size reduction factor and maximum ratio of female-to-male effective population size under different scenarios without post-fission migration and with 2% intervillage male migration.

In the scenarios presented here, males migrate at a rate of 2% between villages. All other scenarios settings and parameter values are the same as for the scenarios in Supplementary Table 4 but the π-based effective population sizes are computed by sampling individuals in all villages.

Scenario / Study	Global π-based male effective population size reduction factor	Global π-based maximum female-to-male effective population size ratio
1	0.89	0.91
2a	0.94	1.01
2b	0.97	1.01
2c	0.90	0.92
2d	1.32	1.39
2e	1.03	1.08
2f	1.01	1.04
2g	1.02	1.04
2h	1.38	1.42

Supplementary Table 9: Global π-based and coalescent-based male effective population size reduction factor and maximum ratio of female-to-male effective population size under different scenarios with post-fission migration and without 2% intervillage male migration.

In the scenarios presented here, there is post-fission migration. All other scenarios settings and parameter values are the same as for the scenarios in Supplementary Table 5 but the π-based effective population sizes are computed by sampling individuals in all villages.

Scenario / Study	Global π-based male effective population size reduction factor	Global π-based maximum female-to-male effective population size ratio
1	0.92	0.91
2a	0.92	0.92
2b	1.09	0.92
2c	0.97	0.90
2d	1.97	1.28
2e	1.89	1.05
2f	1.63	0.96
2g	7.01	1.01
2h	5.65	1.17

Supplementary Table 10: Global π-based and coalescent-based male effective population size reduction factor and maximum ratio of female-to-male effective population size under different scenarios with post-fission migration and 2% intervillage male migration.

In the scenarios presented here, males migrate at a rate of 2% between villages and there is post-fission migration. All other scenarios settings and parameter values are the same as for the scenarios in Supplementary Table 6 but the π-based effective population sizes are computed by sampling individuals in all villages.

Scenario / Study	Global π-based male effective population size reduction factor	Global π-based maximum female-to-male effective population size ratio
1	0.89	0.91
2a	0.92	0.95
2b	1.07	1.04
2c	0.92	0.94
2d	1.63	1.60
2e	1.49	1.54
2f	1.21	1.24
2g	3.28	3.43
2h	2.87	2.80

Supplementary Table 11: Measures of polygyny reported in the literature and in our simulations.

Population	$\frac{\Delta_m}{\Delta_f} \times \frac{\overline{N_f}}{\overline{N_m}}$ *	$\frac{N_m{}^{max}}{\overline{N_m}}$ **
Aché	5.16	2.03
Aka	1.63	2.21
Hazda	1.87	3.52
!Kung	1.61	2.33
Meriam	1.03	3.31
Kipsigis	7.71	6.44
Pimbwe	1.27	2.00
Tsimane	1.54	2.43
Xavante	3.10	6.39
Yanomamö	3.24	7.69
Yomut	0.86	5.86
**Kipsigis-like**	**7.01**	**14.68**

*Ratio of male to female variance in reproductive success (Δ) corrected by the female to male ratio of mean number of children. $\overline{N_m}$ and $\overline{N_f}$ correspond to the mean number of children per male and per female respectively.
**Ratio of maximum number of children for a male to the mean number of children per male.
All figures are computed from Table 1 in Betzig *et al.* [82] except for Kipsigis-like that is obtained from our simulations. In our simulations, the mating model was calibrated so that the ratio of male to female variance in reproductive success corrected by the female to male ratio of mean number of children is similar to that of Kipsigis. Note that the ratio of maximum number of children for a male to the mean number of children per male is higher in the simulated Kipsigis-like population than in the Kipsigis population. The Kipsigis-like values for variance in reproductive success and the maximum number of children for a male were obtained by computing means across all generations and all replicates.

Supplementary figures

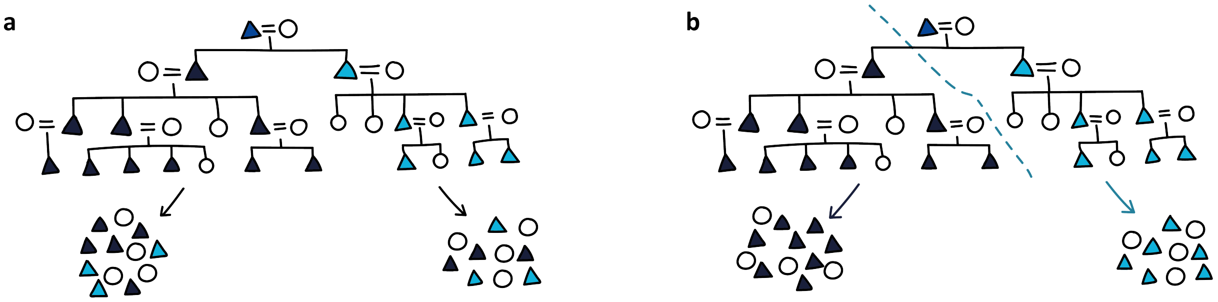

Supplementary Figure 1: Fission types

Triangles represent males while circles represent females. In this representation, all males are affiliated through the male line (patrilineal descent group). **a**: Random fission. **b**: Lineal fission, the most related males cluster together in the newly formed descent groups.

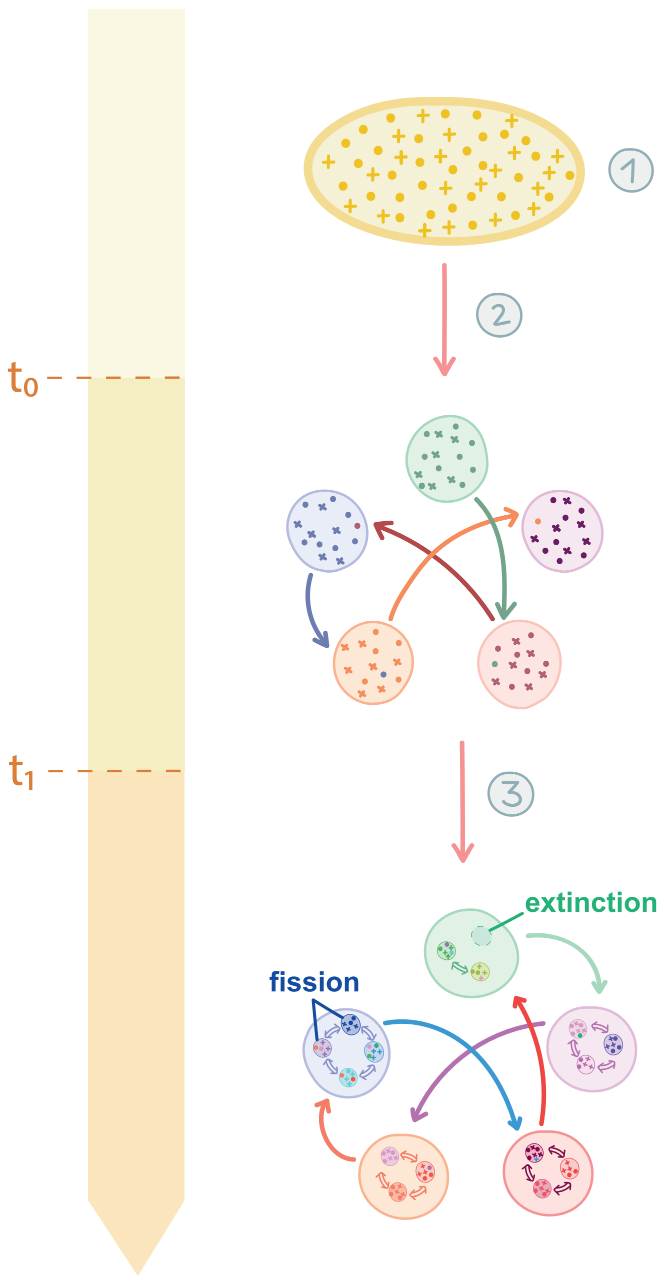

Supplementary Figure 2: Description of the two transitions scenario

1: panmictic population of constant size. **2**: at t_0, the population splits into 5 villages with bilateral descent and patrilocal, multilocal or matrilocal residence rule. The population begins to grow at an exponential rate ($r = 0.01$). **3**: at t_1, there is a transition to a patrilineal and patrilocal system with the same settings as in scenario 2g, i.e. lineal fission, variance in reproductive success between patrilineal groups, no violence (see legend of Figure 2 for parameter values).

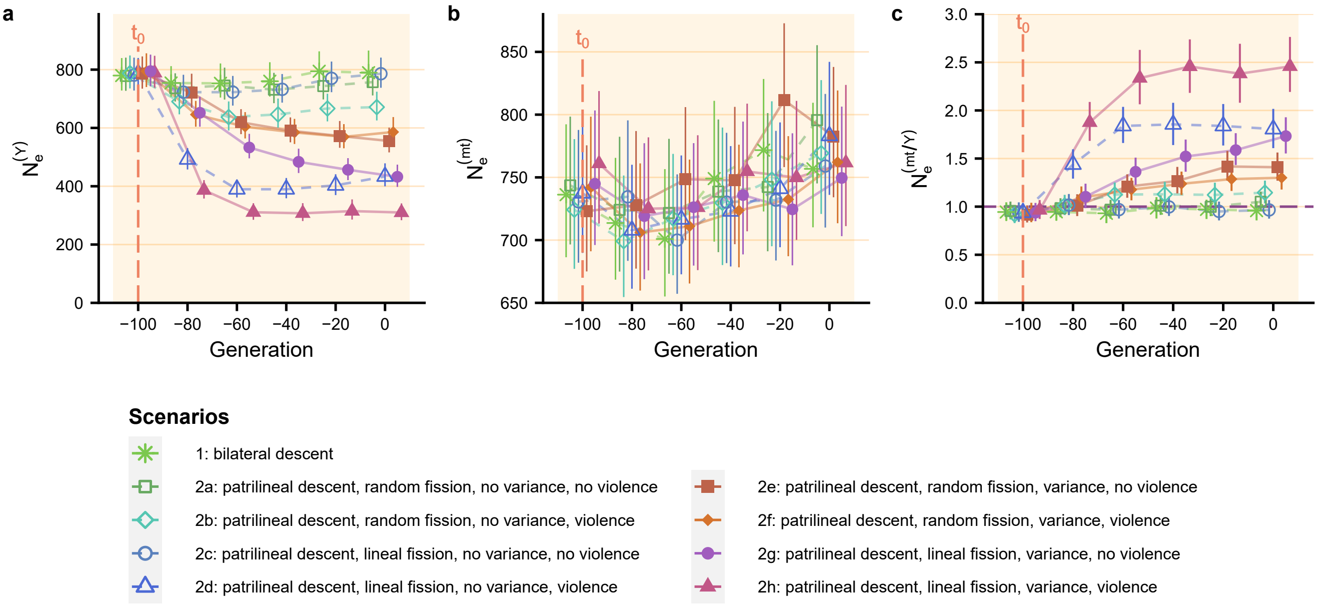

Supplementary Figure 3: Change in π-based male and female effective population sizes over time for scenarios without post-fission migration and with 2% intervillage male migration

In the scenarios presented here, male migrate at a rate of 2% between villages. All other parameters are identical to those in Figure 2. π-based male effective population size (**a**), female effective population size (**b**) and female-to-male N_e ratios (**c**) averaged over 200 replicates are shown every 20 generations with the 95% confidence interval. Values of means and their 95% confidence intervals are provided in the Source Data file.

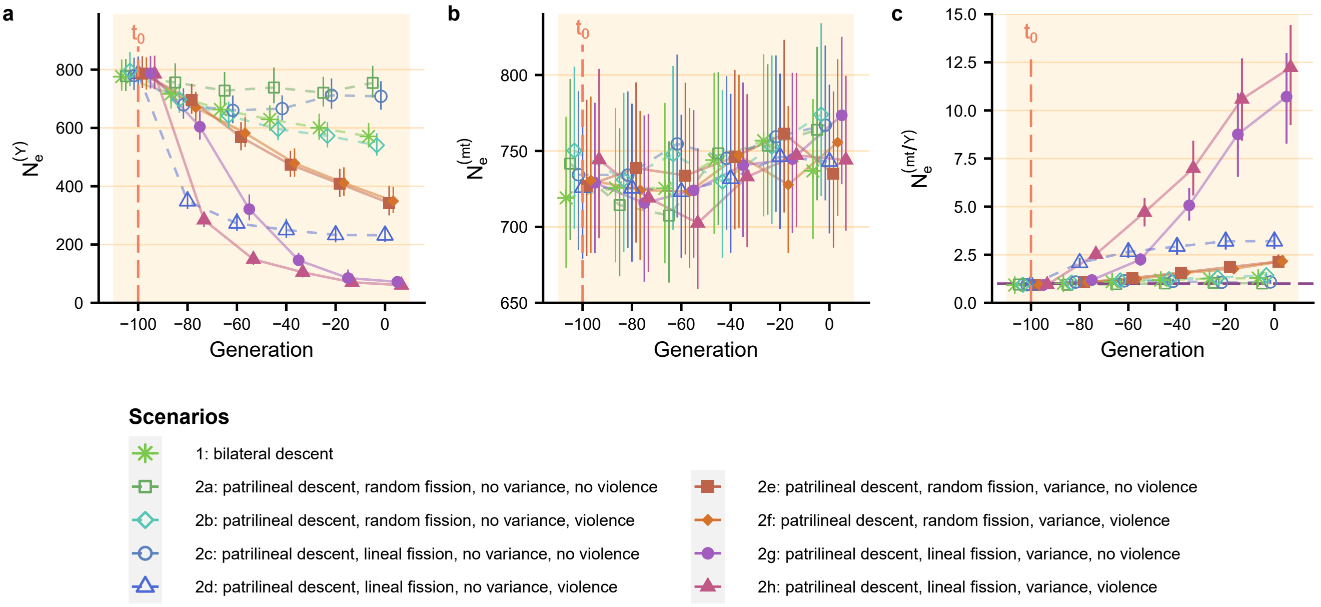

Supplementary Figure 4: Change in π-based male and female effective population sizes over time for scenarios with post-fission migration and without 2% intervillage male migration

In the scenarios presented here, there is post-fission migration of descent groups between villages. All other parameters are identical to those in Figure 2. π-based male effective population size (**a**), female effective population size (**b**) and female-to-male N_e ratios (**c**) averaged over 200 replicates are shown every 20 generations with the 95% confidence interval. Values of means and their 95% confidence intervals are provided in the Source Data file.

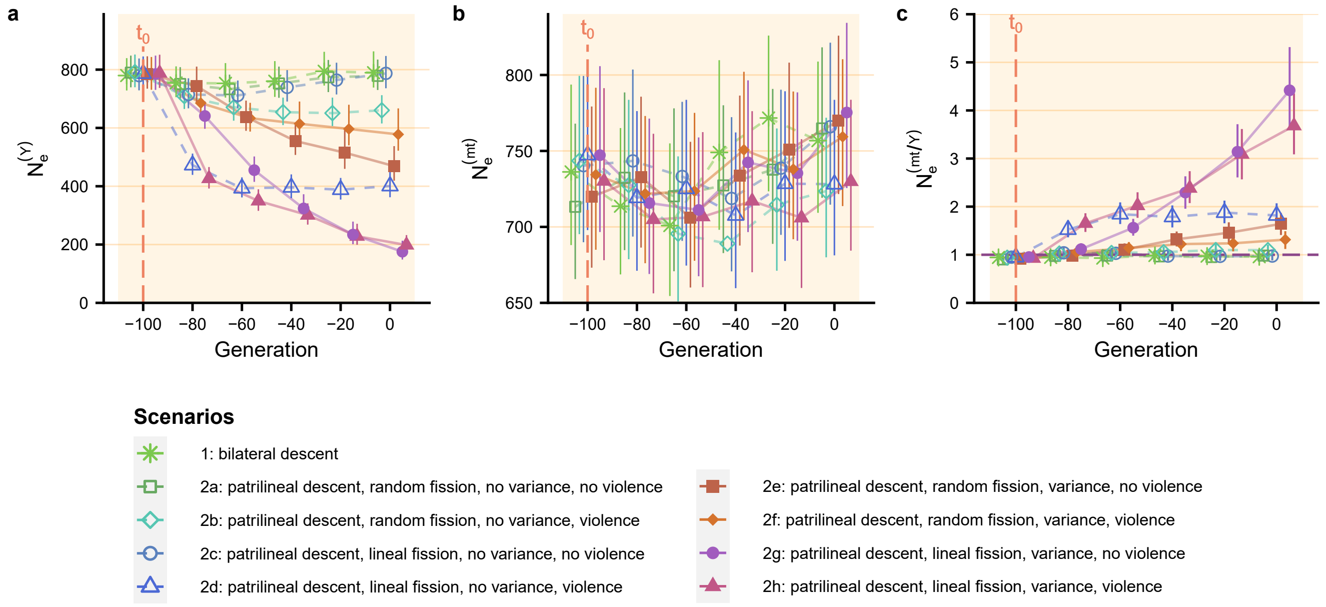

Supplementary Figure 5: Change in π-based male and female effective population sizes over time for scenarios with post-fission and 2% intervillage male migration

In the scenarios presented here, male migrate at a rate of 2% between villages and there is post-fission migration of descent groups between villages. All other parameters are identical to those in Figure 2. π-based male effective population size (**a**), female effective population size (**b**) and female-to-male N_e ratios (**c**) averaged over 200 replicates are shown every 20 generations with the 95% confidence interval. Values of means and their 95% confidence intervals are provided in the Source Data file.

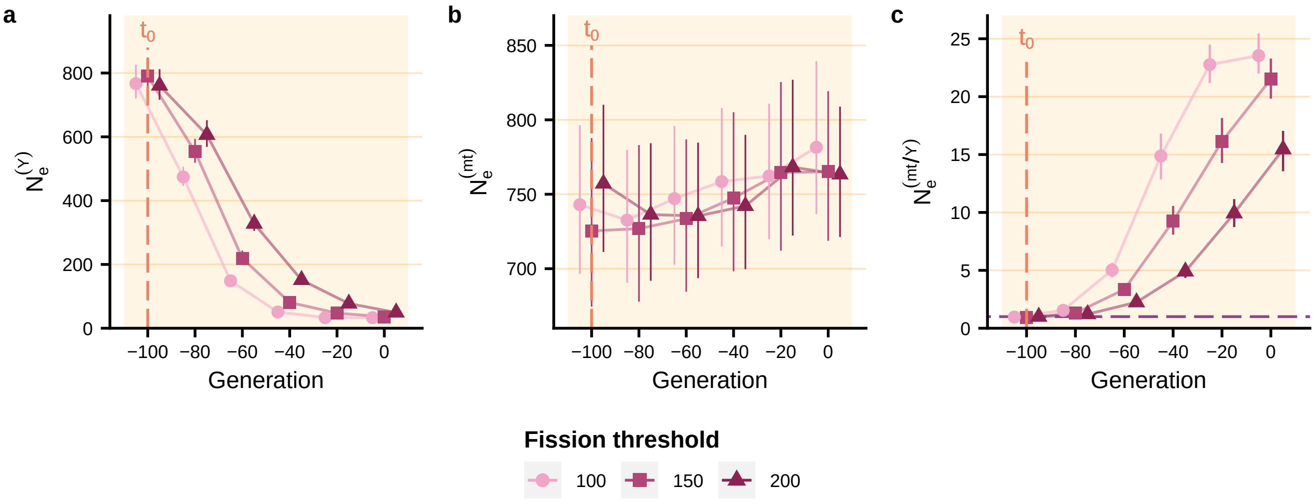

Supplementary Figure 6: Change in π-based male and female effective population sizes over time for different fission thresholds

Patrilocal residence and patrilineal descent (with settings of scenario 2g, i.e. lineal fission, variance in reproductive success between descent groups and no violence) are introduced at t_0, 100 generations before present, after a phase of panmixia. See legend of Figure 2 for parameter values (except for the parameter controlling the fission threshold, which varies between 100 and 200). π-based male effective population size (**a**), female effective population size (**b**), and female-to-male effective population size ratio (**c**) averaged over 200 replicates are shown every 20 generations with the 95% confidence interval. Values of means and their 95% confidence intervals are provided in the Source Data file.

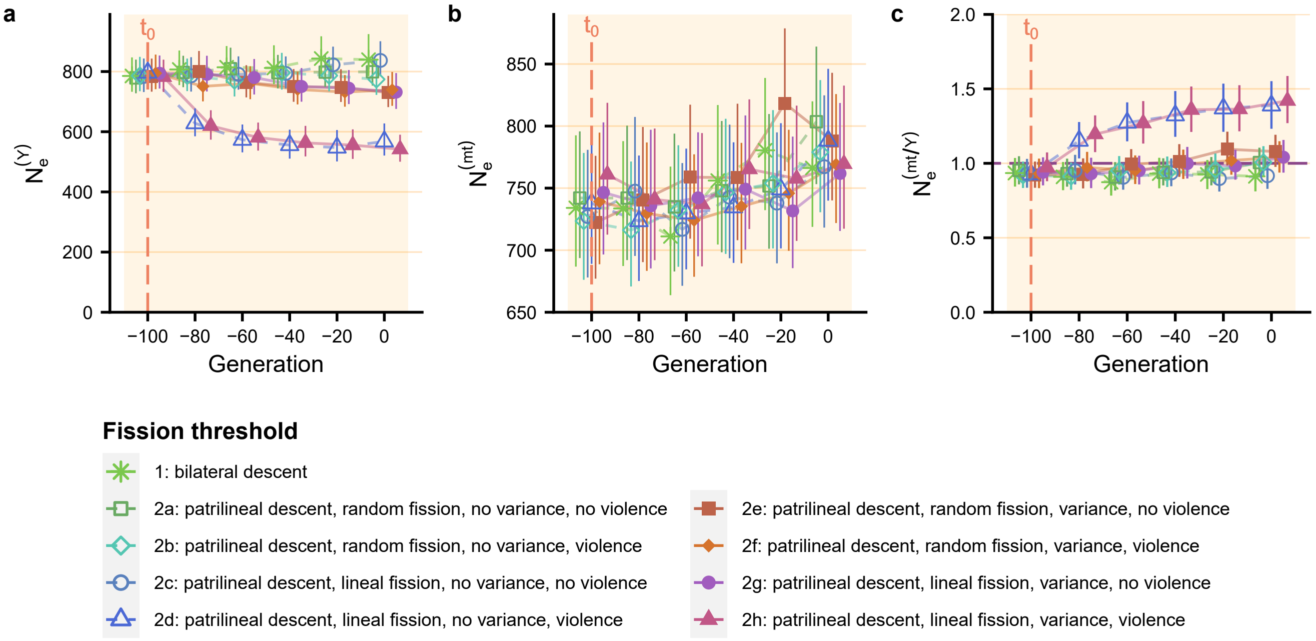

Supplementary Figure 7: Change in global π-based male and female effective population size over time under scenarios without post-fission migration or intervillage male migration

Patrilocal residence and the descent rule of interest are introduced at t_0, 100 generations before present, after a phase of panmixia. Scenarios settings and parameter values are the same as for the scenarios in Figure 2 but the π-based effective population sizes are computed by sampling individuals in all villages. Global π-based male effective population size (**a**), female effective population size (**b**) and female-to-male N_e ratios (**c**) averaged over 200 replicates are shown every 20 generations with the 95% confidence interval. Values of means and their 95% confidence intervals are provided in the Source Data file.

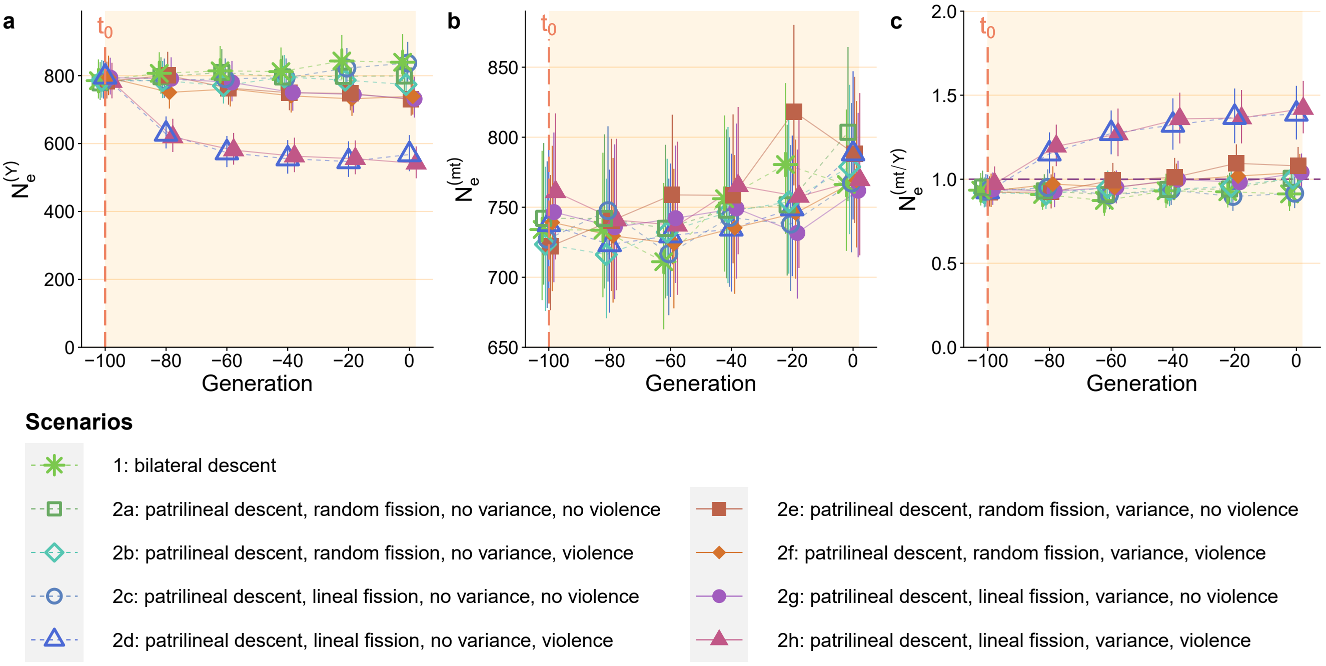

Supplementary Figure 8: Change in global π-based male and female effective population size over time under scenarios without post-fission migration and with 2% intervillage male migration

Patrilocal residence and the descent rule of interest are introduced at t_0, 100 generations before present, after a phase of panmixia. Scenarios settings and parameter values are the same as for the scenarios in Supplementary Fig. 3 but the π-based effective population sizes are computed by sampling individuals in all villages. Global π-based male effective population size (**a**), female effective population size (**b**) and female-to-male N_e ratios (**c**) averaged over 200 replicates are shown every 20 generations with the 95% confidence interval. Values of means and their 95% confidence intervals are provided in the Source Data file.

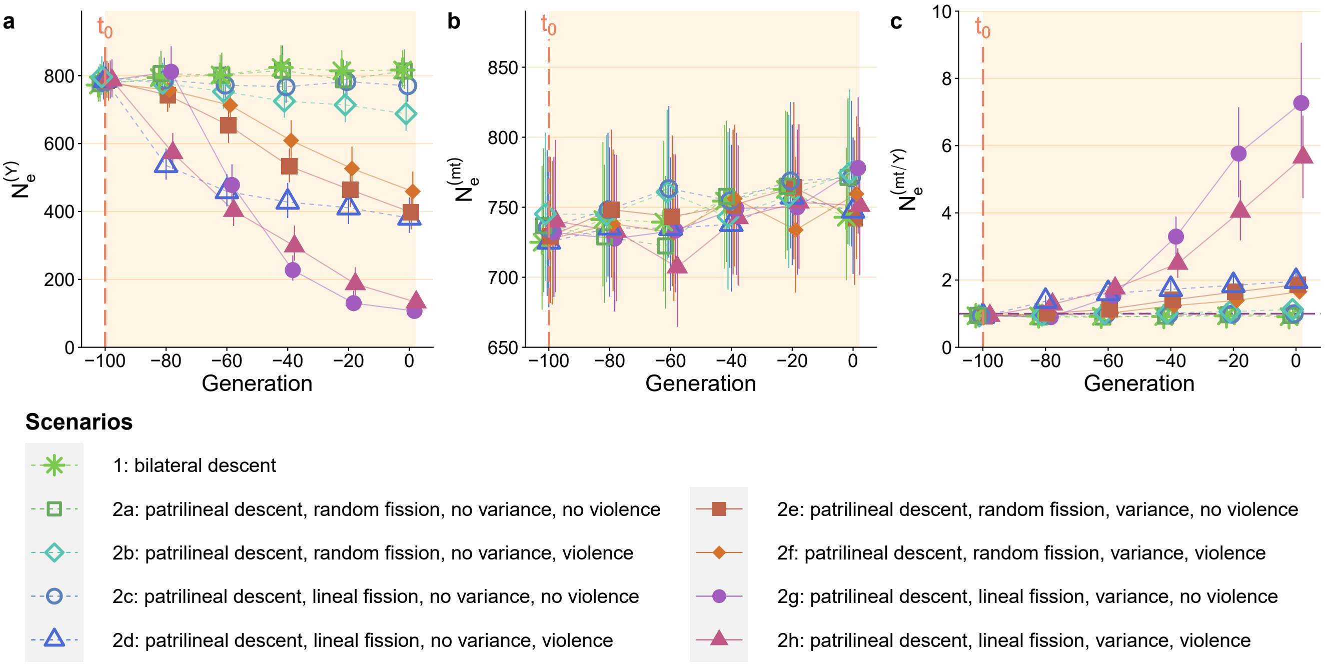

Supplementary Figure 9: Change in global π-based male and female effective population size over time under scenarios with post-fission migration and without 2% intervillage male migration

Patrilocal residence and the descent rule of interest are introduced at t_0, 100 generations before present, after a phase of panmixia. Scenarios settings and parameter values are the same as for the scenarios in Supplementary Fig. 4 but the π-based effectives sizes are computed by sampling individuals in all villages. Global π-based male effective population size (**a**), female effective population size (**b**) and female-to-male N_e ratios (**c**) averaged over 200 replicates are shown every 20 generations with the 95% confidence interval. Values of means and their 95% confidence intervals are provided in the Source Data file.

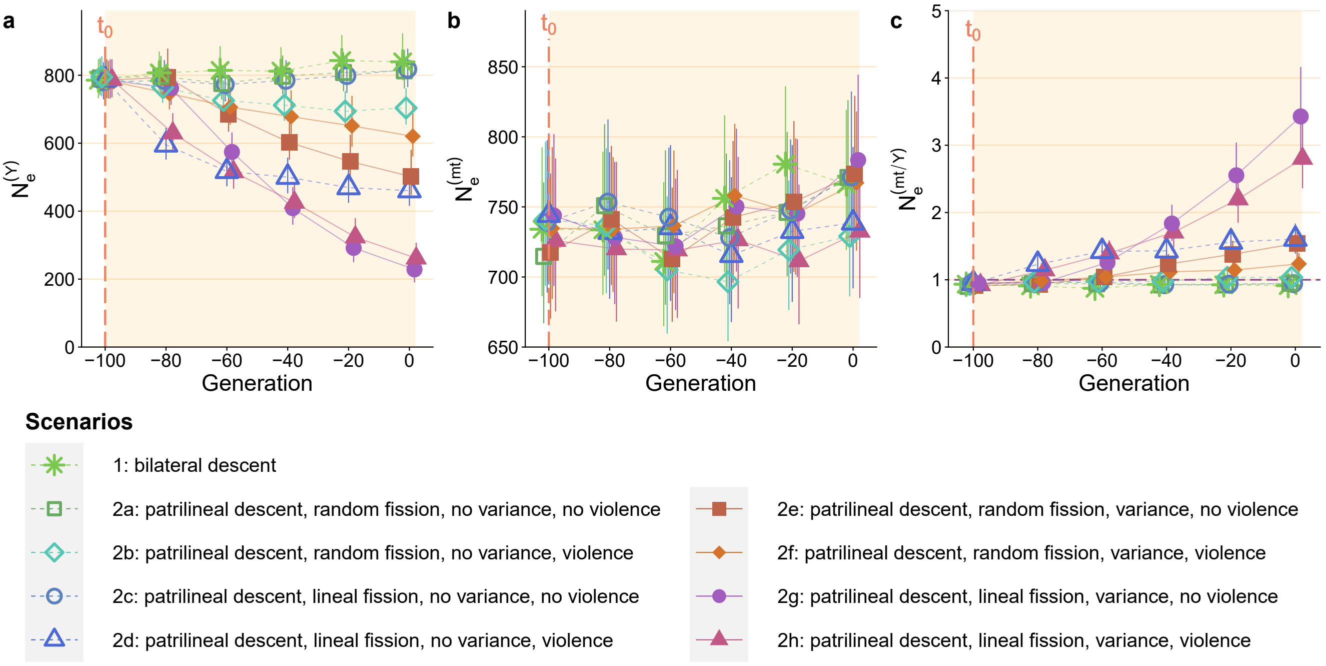

Supplementary Figure 10: Change in global π-based male and female effective population size over time under scenarios with post-fission migration and 2% intervillage male migration

Patrilocal residence and the descent rule of interest are introduced at t_0, 100 generations before present, after a phase of panmixia. Scenarios settings and parameter values are the same as for the scenarios in Supplementary Fig. 5 but the π-based effective population sizes are computed by sampling individuals in all villages. Global π-based male effective population size (**a**), female effective population size (**b**) and female-to-male N_e ratios (**c**) averaged over 200 replicates are shown every 20 generations with the 95% confidence interval. Values of means and their 95% confidence intervals are provided in the Source Data file.

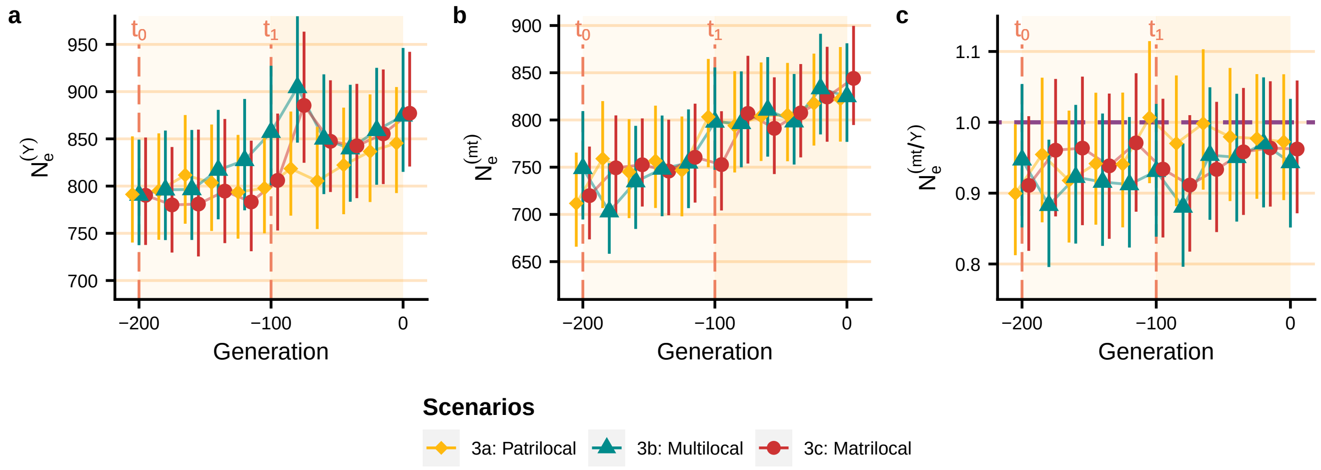

Supplementary Figure 11: Change in global π-based male and female effective population size over time under the two transitions scenario

Scenarios settings and parameter values are the same as for the scenarios in Figure 3 but the π-based effective population sizes are computed by sampling individuals in all villages. Global π-based male effective population size (**a**), female effective population size (**b**) and female-to-male N_e ratios (**c**) averaged over 200 replicates are shown every 20 generations with the 95% confidence interval. Values of means and their 95% confidence intervals are provided in the Source Data file.

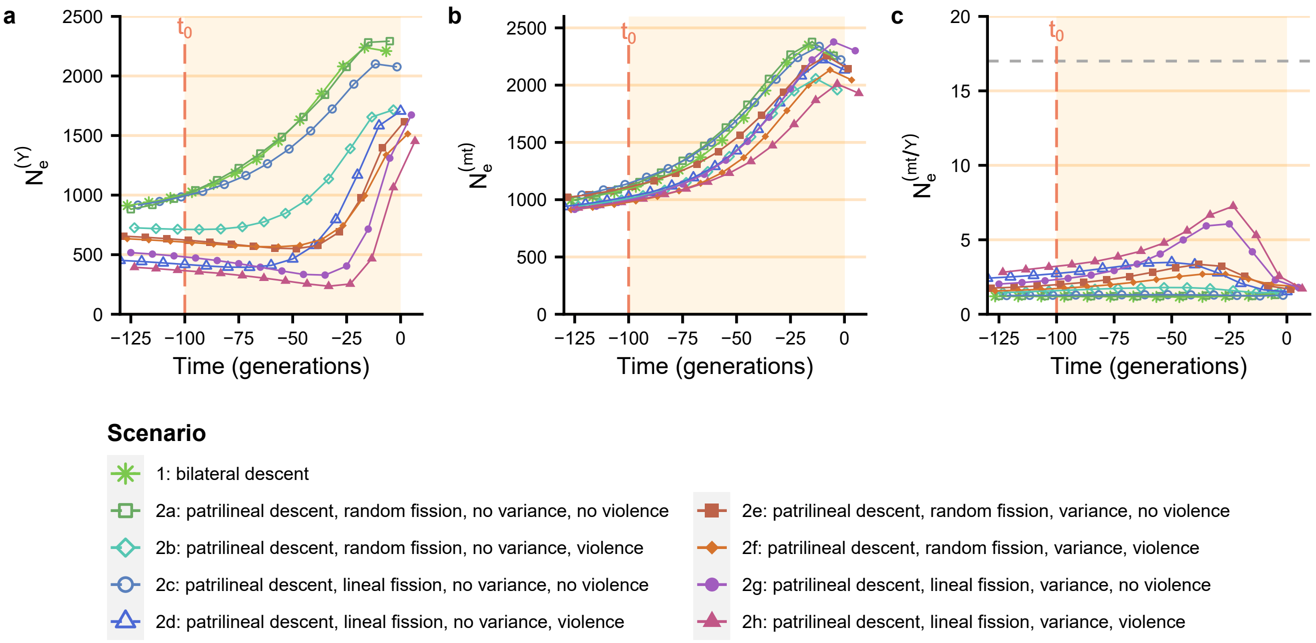

Supplementary Figure 12: Change in coalescent-based male and female effective population sizes over time under different scenarios without post-fission migration and with 2% intervillage male migration

Patrilocal residence and the descent rule of interest are introduced at t_0, 100 generations before present, after a phase of panmixia. Scenarios settings and parameter values are the same as for the scenarios in Supplementary Fig. 3 but the coalescent-based effective population sizes are computed by sampling individuals in all villages. Average Bayesian skyline plots of male effective population size (**a**), female effective population size (**b**), and female-to-male N_e ratio (**c**) over 200 replicates were plotted for each scenario. Values of means are provided in the Source Data file.

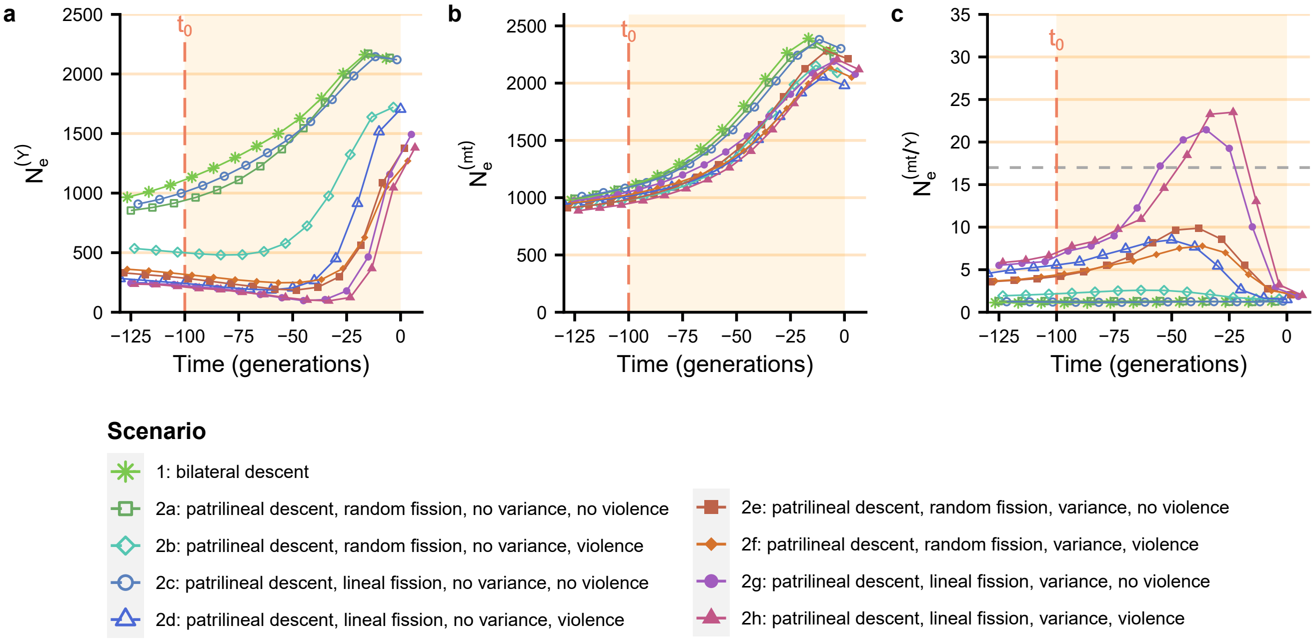

Supplementary Figure 13: Change in coalescent-based male and female effective population sizes over time under different scenarios with post-fission migration and without 2% intervillage male migration

Patrilocal residence and the descent rule of interest are introduced at t_0, 100 generations before present, after a phase of panmixia. Scenarios settings and parameter values are the same as for the scenarios in Supplementary Fig. 4 but the coalescent-based effective population sizes are computed by sampling individuals in all villages. Average Bayesian skyline plots of male effective population size (**a**), female effective population size (**b**), and female-to-male N_e ratio (**c**) over 200 replicates were plotted for each scenario. Values of means are provided in the Source Data file.

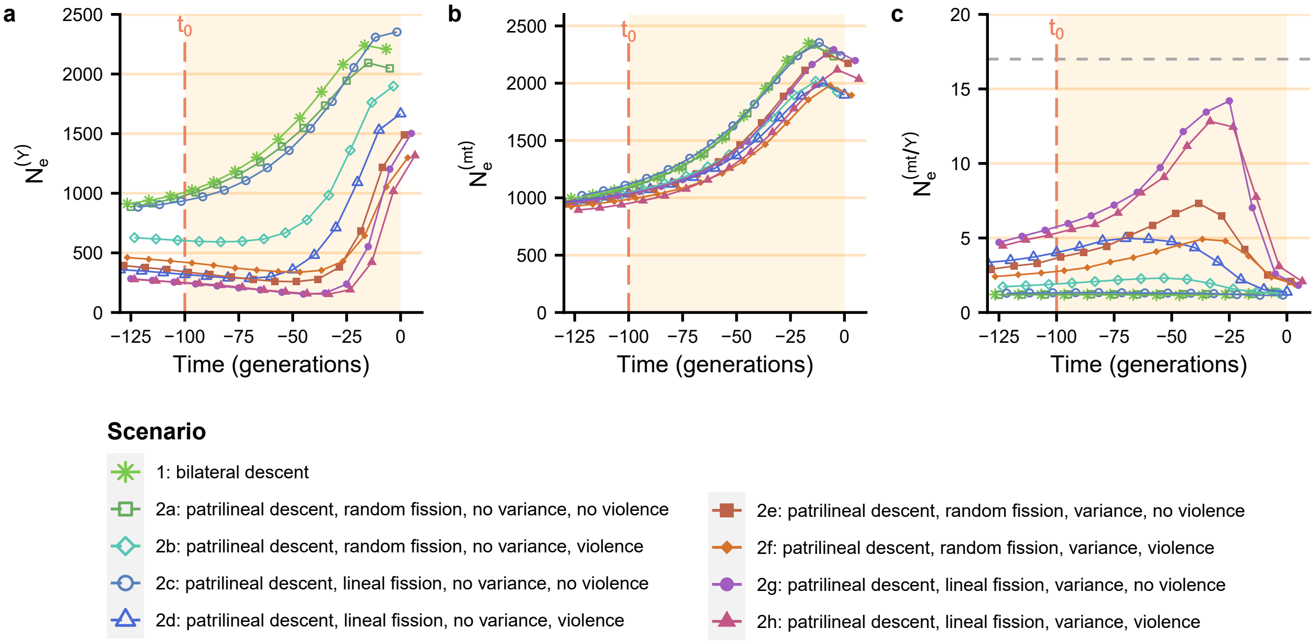

Supplementary Figure 14: Change in coalescent-based male and female effective population sizes over time under different scenarios with post-fission migration and 2% intervillage male migration

Patrilocal residence and the descent rule of interest are introduced at t_0, 100 generations before present, after a phase of panmixia. Scenarios settings and parameter values are the same as for the scenarios in Supplementary Fig. 5 but the coalescent-based effective population sizes are computed by sampling individuals in all villages. Average Bayesian skyline plots of male effective population size (**a**), female effective population size (**b**), and female-to-male N_e ratio (**c**) over 200 replicates were plotted for each scenario. Values of means are provided in the Source Data file.

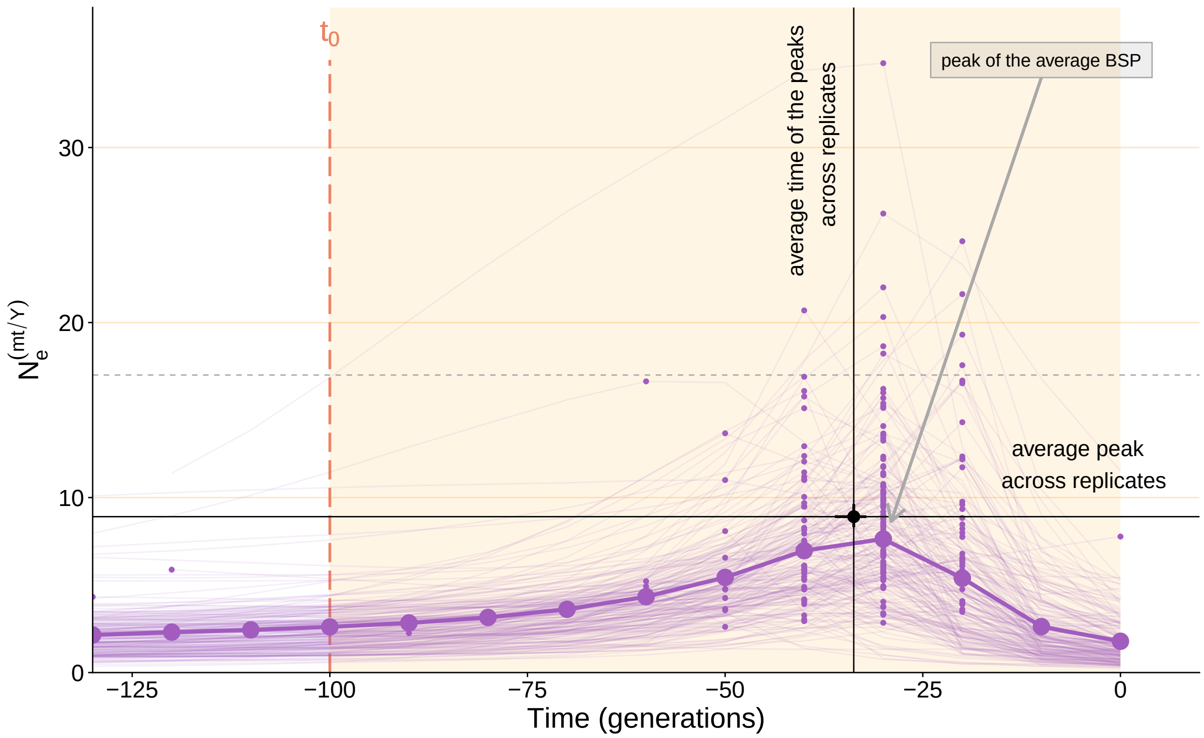

Supplementary Figure 15: Comparison of two estimators of the coalescent-based maximum female-to-male effective population size ratio

The female-to-male N_e ratio can be computed in different ways. In Table 2 and Supplementary Tables 4-6, we report the peak of the average Baysesian Skyline Plot (BSP) for consistency with Figure 5 and Supplementary Fig. 12-14, which show the average BSP. Because each BSP (for each replicate) peaks at different times, this value is underestimated compared to the average of the peaks across replicates shown in the figure. Patrilocality and patrilineality are introduced at t_0, 100 generations before present, after a phase of panmixia, following the settings of scenario 2g. See legend of Figure 2 for parameter values. The light purple curves correspond to the Bayesian skyline plots (BSPs) of each replicate. The bold purple curve is the average BSP over all replicates. The small purple points correspond to the peak of the BSP of each replicate. Values of means are provided in the Source Data file.

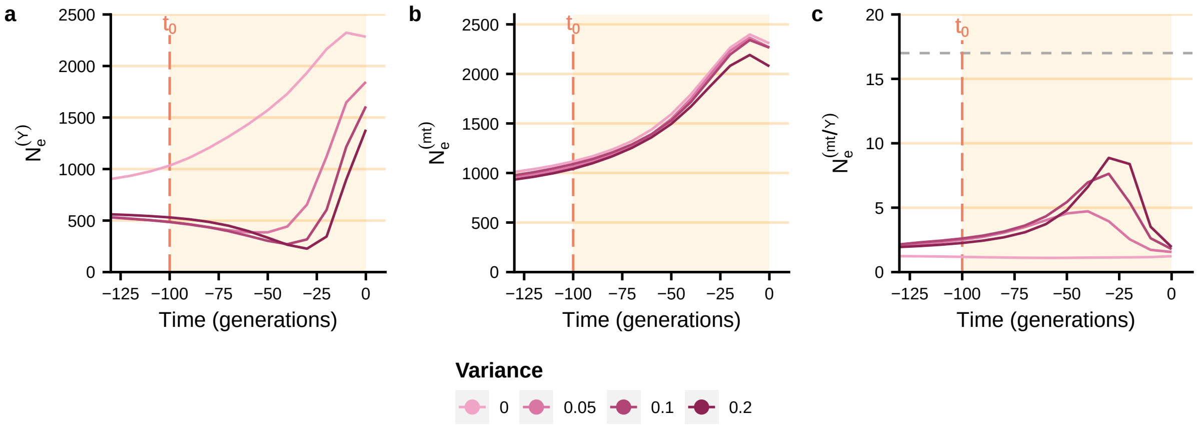

Supplementary Figure 16: Change in coalescent-based male and female effective population sizes for a range of variances in reproductive success between descent groups

Patrilocal residence and patrilineal descent (with settings of scenario 2g, i.e. lineal fission, variance in reproductive success between descent groups and no violence) are introduced at t_0, 100 generations before present, after a phase of panmixia. See legend of Figure 2 for parameter values (except for the parameter controlling the variance in reproductive success between descent groups, which varies between 0 and 0.2). Average Bayesian skyline plots of male effective population size (**a**), female effective population size (**b**), and female-to-male N_e ratio (**c**) over 200 replicates were plotted for each scenario. Values of means are provided in the Source Data file.

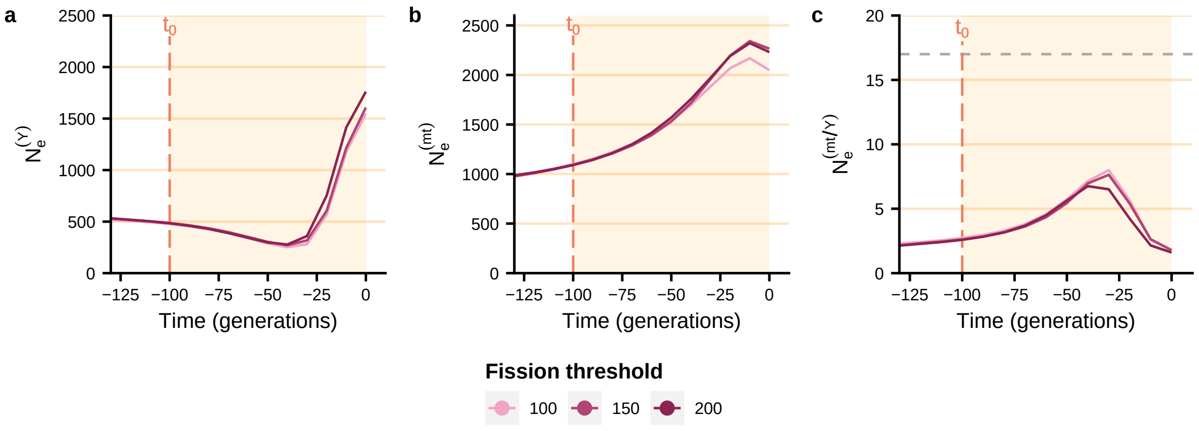

Supplementary Figure 17: Change in coalescent-based male and female effective population sizes for different fission thresholds

Patrilocal residence and patrilineal descent (with settings of scenario 2g, i.e. lineal fission, variance in reproductive success between descent groups and no violence) are introduced at t_0, 100 generations before present, after a phase of panmixia. See legend of Figure 2 for parameter values (except for the parameter controlling the fission threshold, which varies between 100 and 200). Average Bayesian skyline plots of male effective population size (**a**), female effective population size (**b**), and female-to-male N_e ratio (**c**) over 200 replicates were plotted for each scenario. Values of means are provided in the Source Data file.

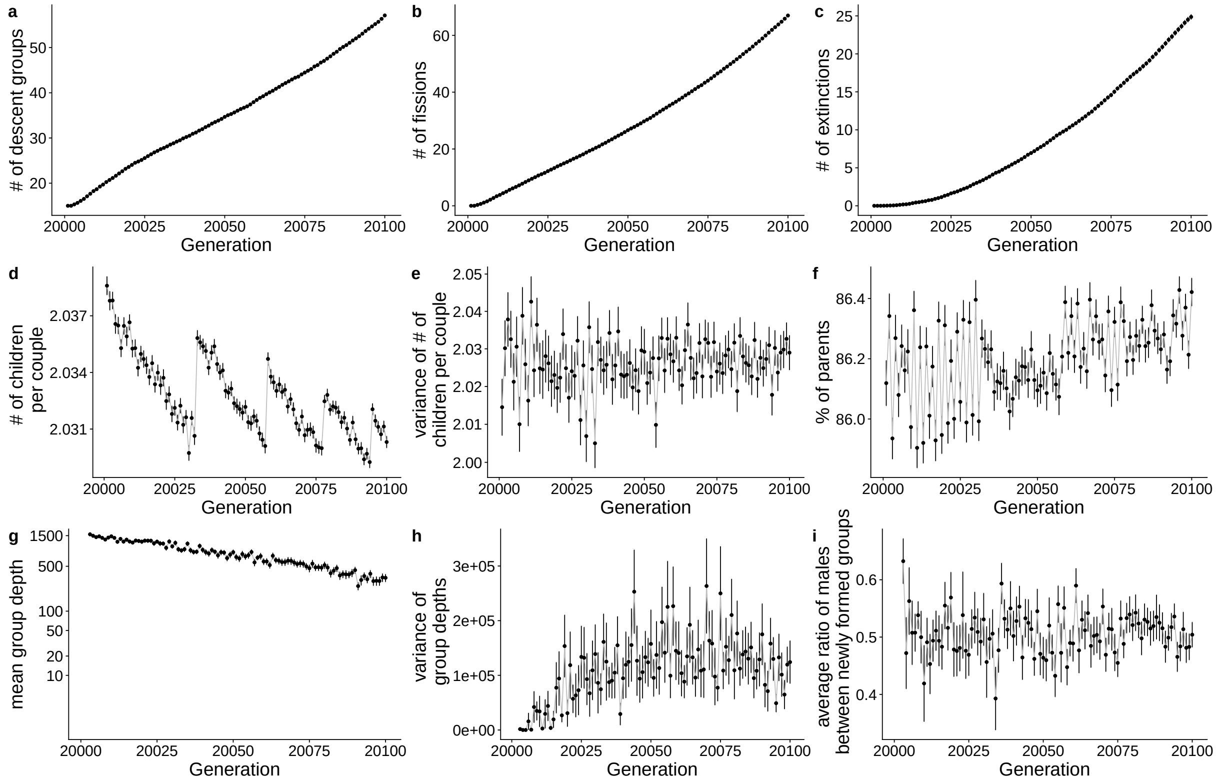

Supplementary Figure 18: Change in demographic metrics over time for simulations of the scenario 2a without post-fission migration or 2% intervillage migration

Means and their 95% confidence intervals calculated on all replicates are shown every two generations. **a**: average number of descent groups. **b**: average cumulated number of fissions. **c**: average cumulated number of extinctions. **d**: average number of children per couple (without taking into account single individuals). **e**: average variance of number of children per couple (without taking into account single individuals). **f**: average percentage of female having children. **g**: average mean number of generations to the most recent common ancestors of descent groups. **h**: average variance of number of generations to the most recent common ancestors of descent groups. **i**: average ratio of number of individuals between resulting groups after a fission event.

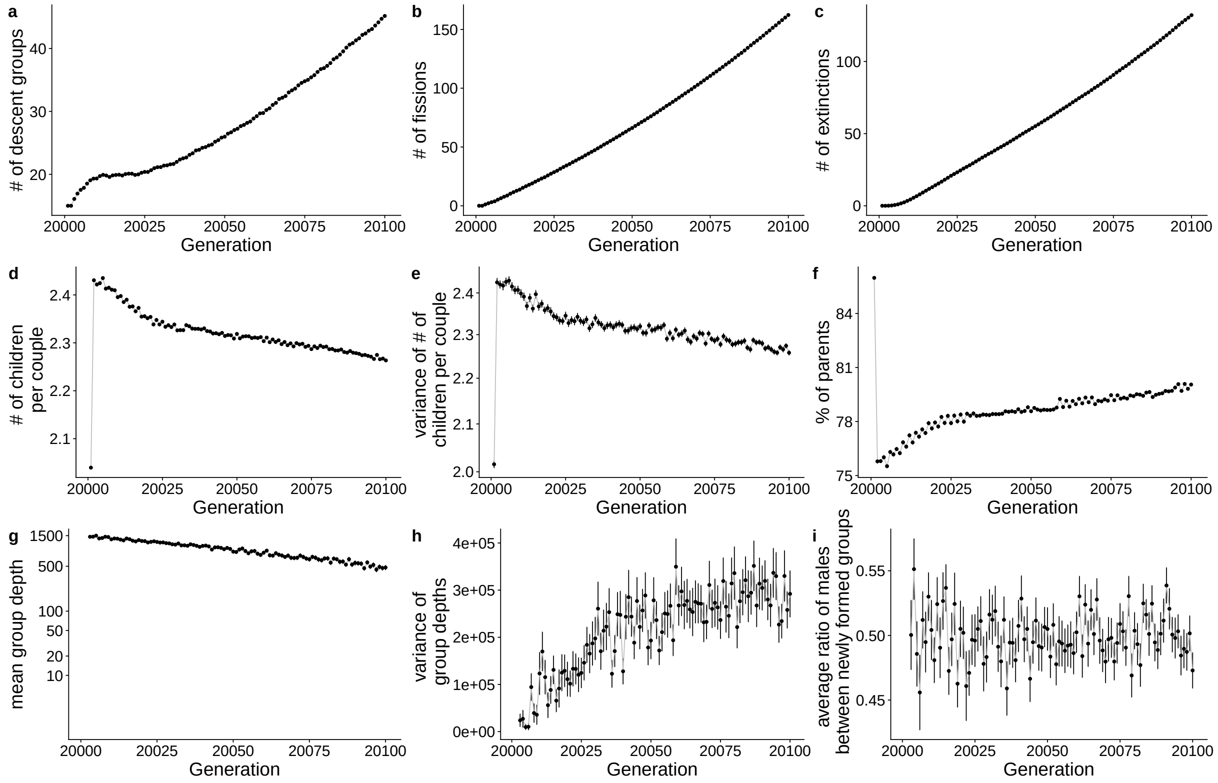

Supplementary Figure 19: Change in demographic metrics over time for simulations of the scenario 2b without post-fission migration or 2% intervillage migration

Means and their 95% confidence intervals calculated on all replicates are shown every two generations. **a**: average number of descent groups. **b**: average cumulated number of fissions. **c**: average cumulated number of extinctions. **d**: average number of children per couple (without taking into account single individuals). **e**: average variance of number of children per couple (without taking into account single individuals). **f**: average percentage of female having children. **g**: average mean number of generations to the most recent common ancestors of descent groups. **h**: average variance of number of generations to the most recent common ancestors of descent groups. **i**: average ratio of number of individuals between resulting groups after a fission event.

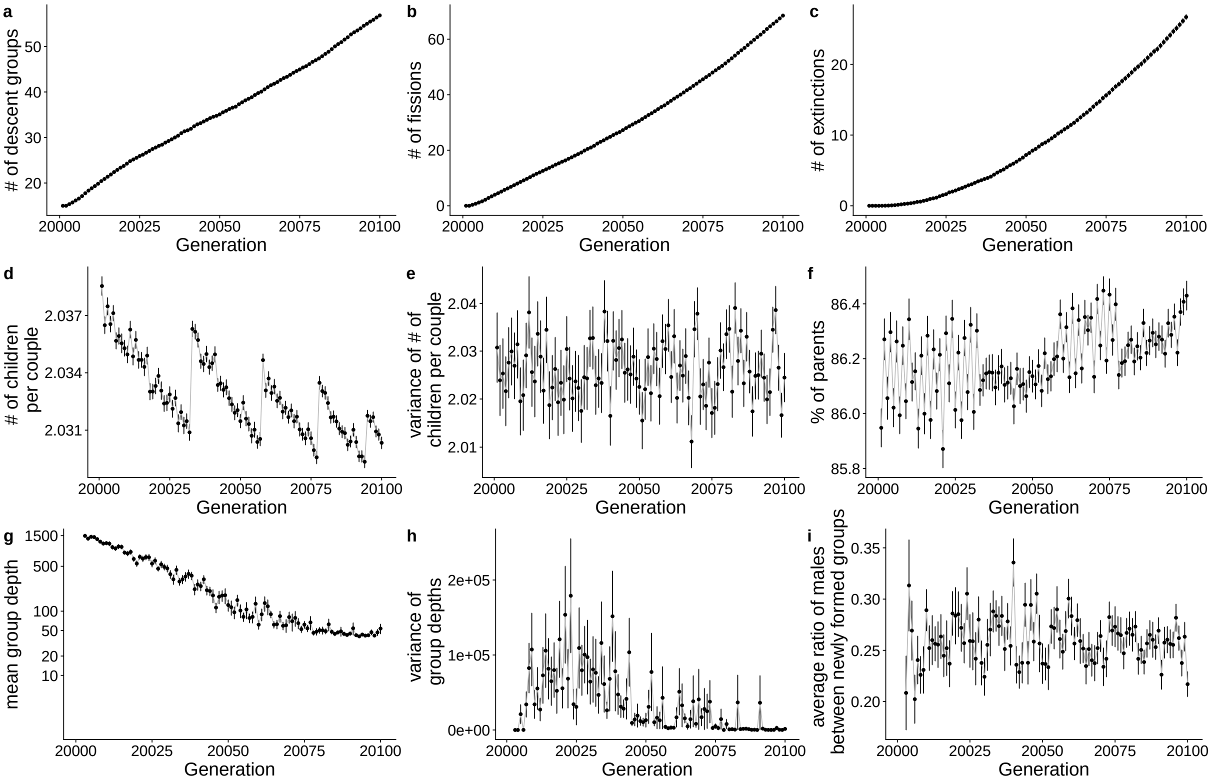

Supplementary Figure 20: Change in demographic metrics over time for simulations of the scenario 2c without post-fission migration or 2% intervillage migration

Means and their 95% confidence intervals calculated on all replicates are shown every two generations. **a**: average number of descent groups. **b**: average cumulated number of fissions. **c**: average cumulated number of extinctions. **d**: average number of children per couple (without taking into account single individuals). **e**: average variance of number of children per couple (without taking into account single individuals). **f**: average percentage of female having children. **g**: average mean number of generations to the most recent common ancestors of descent groups. **h**: average variance of number of generations to the most recent common ancestors of descent groups. **i**: average ratio of number of individuals between resulting groups after a fission event.

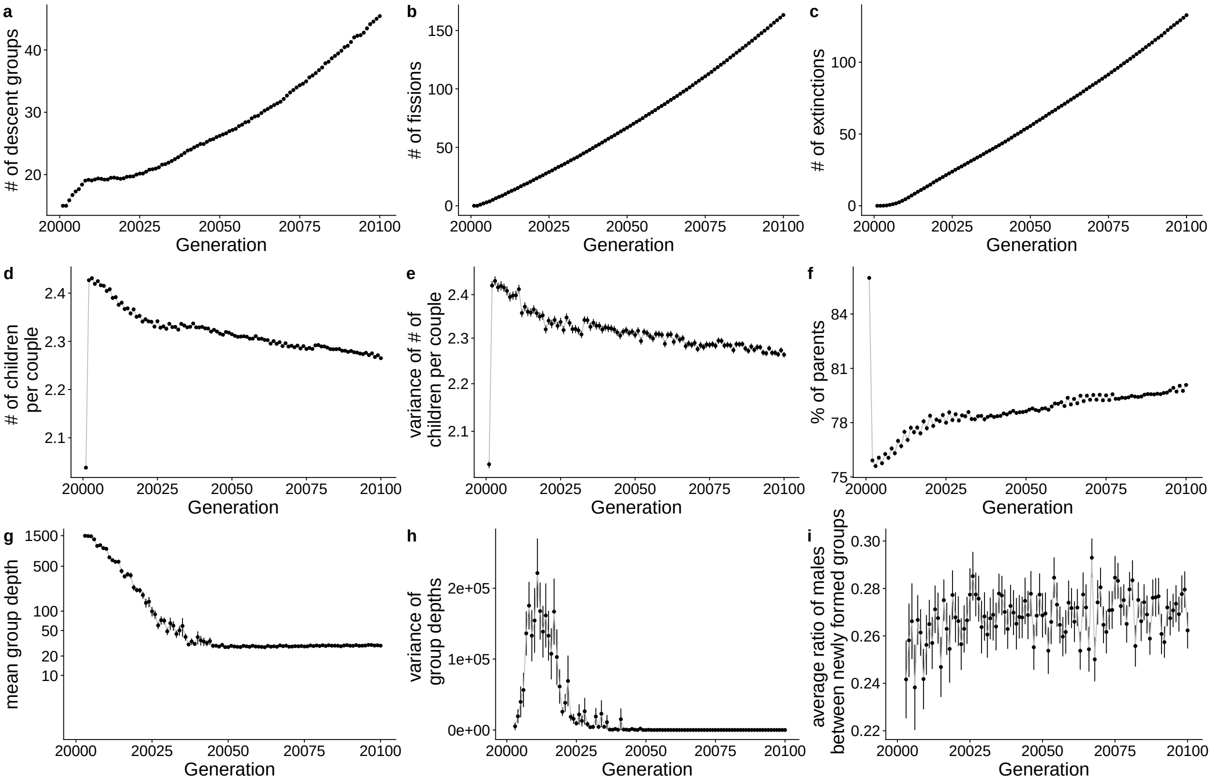

Supplementary Figure 21: Change in demographic metrics over time for simulations of the scenario 2d without post-fission migration or 2% intervillage migration

Means and their 95% confidence intervals calculated on all replicates are shown every two generations. **a**: average number of descent groups. **b**: average cumulated number of fissions. **c**: average cumulated number of extinctions. **d**: average number of children per couple (without taking into account single individuals). **e**: average variance of number of children per couple (without taking into account single individuals). **f**: average percentage of female having children. **g**: average mean number of generations to the most recent common ancestors of descent groups. **h**: average variance of number of generations to the most recent common ancestors of descent groups. **i**: average ratio of number of individuals between resulting groups after a fission event.

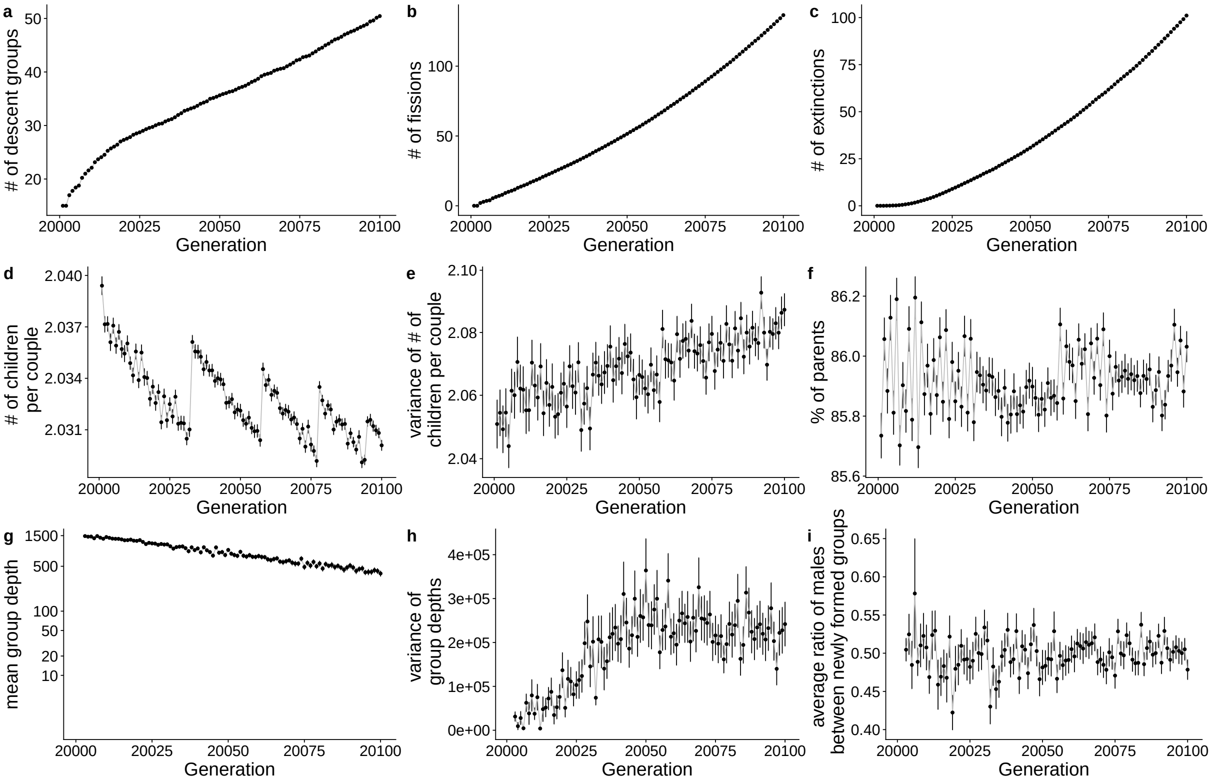

Supplementary Figure 22: Change in demographic metrics over time for simulations of the scenario 2e without post-fission migration or 2% intervillage migration

Means and their 95% confidence intervals calculated on all replicates are shown every two generations. **a**: average number of descent groups. **b**: average cumulated number of fissions. **c**: average cumulated number of extinctions. **d**: average number of children per couple (without taking into account single individuals). **e**: average variance of number of children per couple (without taking into account single individuals). **f**: average percentage of female having children. **g**: average mean number of generations to the most recent common ancestors of descent groups. **h**: average variance of number of generations to the most recent common ancestors of descent groups. **i**: average ratio of number of individuals between resulting groups after a fission event.

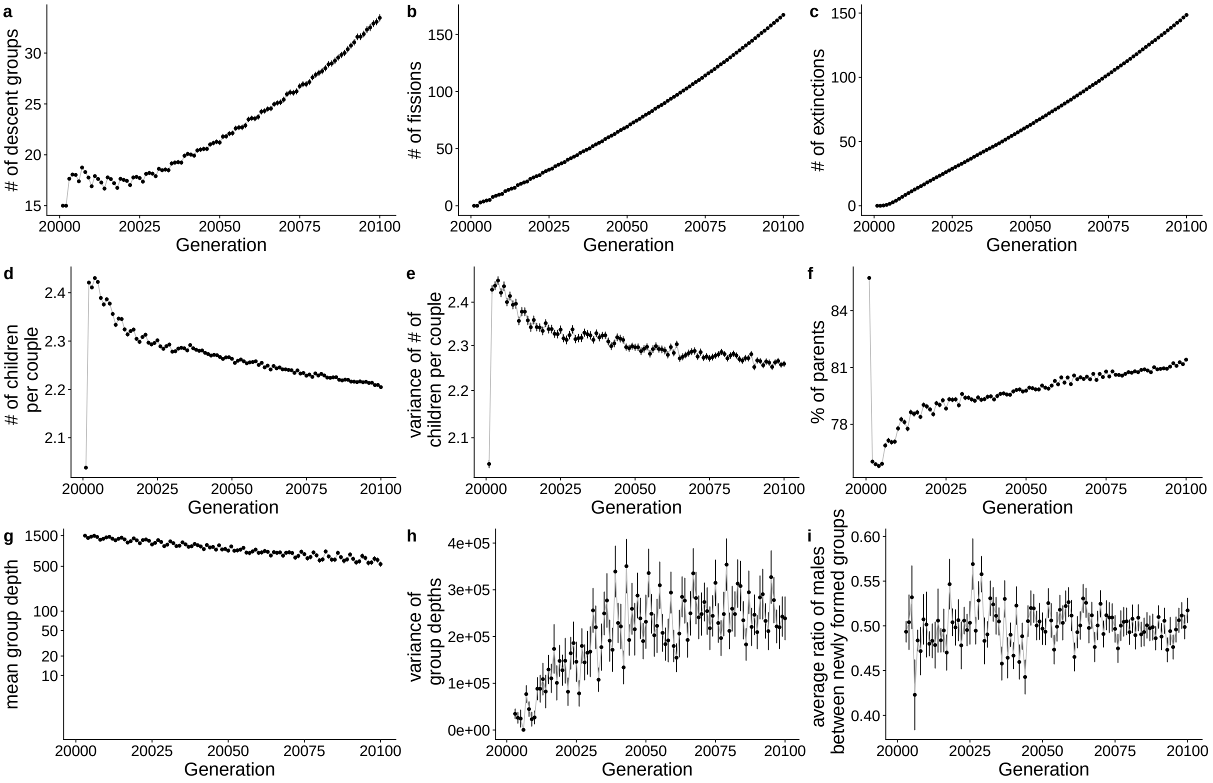

Supplementary Figure 23: Change in demographic metrics over time for simulations of the scenario 2f without post-fission migration or 2% intervillage migration

Means and their 95% confidence intervals calculated on all replicates are shown every two generations. **a**: average number of descent groups. **b**: average cumulated number of fissions. **c**: average cumulated number of extinctions. **d**: average number of children per couple (without taking into account single individuals). **e**: average variance of number of children per couple (without taking into account single individuals). **f**: average percentage of female having children. **g**: average mean number of generations to the most recent common ancestors of descent groups. **h**: average variance of number of generations to the most recent common ancestors of descent groups. **i**: average ratio of number of individuals between resulting groups after a fission event.

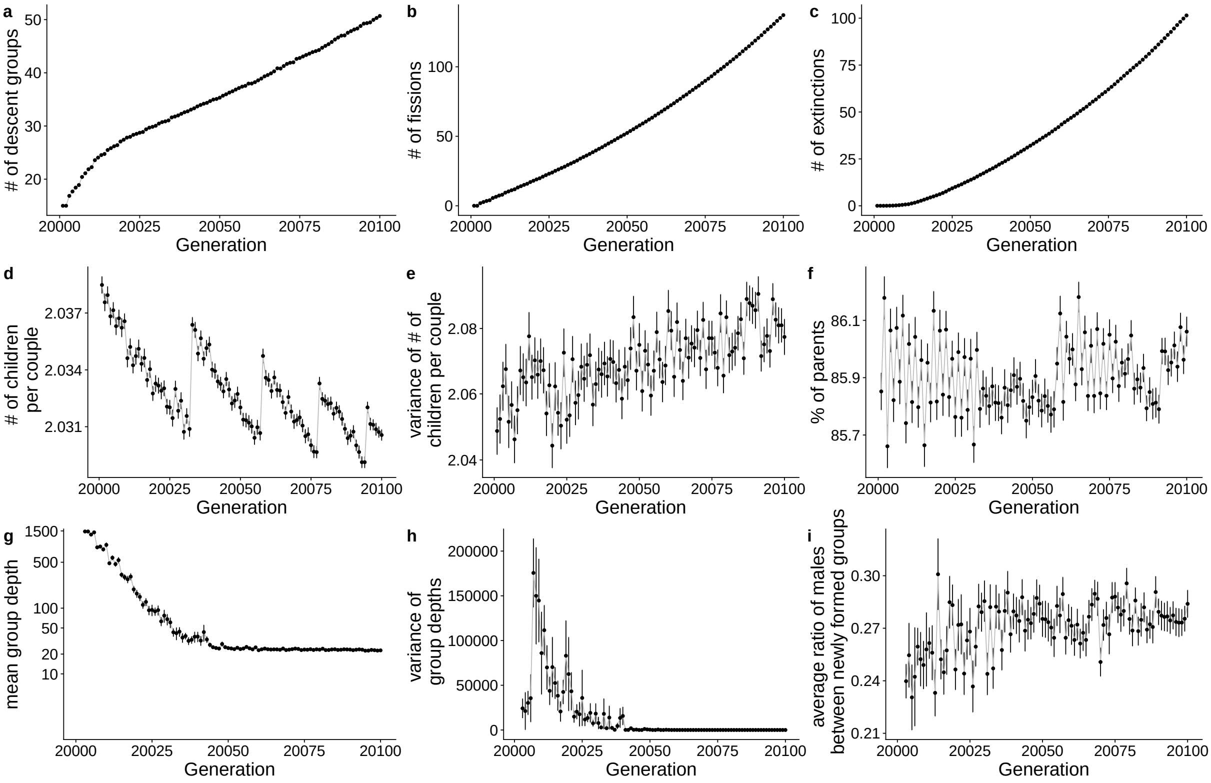

Supplementary Figure 24: Change in demographic metrics over time for simulations of the scenario 2g without post-fission migration or 2% intervillage migration

Means and their 95% confidence intervals calculated on all replicates are shown every two generations. **a**: average number of descent groups. **b**: average cumulated number of fissions. **c**: average cumulated number of extinctions. **d**: average number of children per couple (without taking into account single individuals). **e** average variance of number of children per couple (without taking into account single individuals). **f**: average percentage of female having children. **g**: average mean number of generations to the most recent common ancestors of descent groups. **h**: average variance of number of generations to the most recent common ancestors of descent groups. **i**: average ratio of number of individuals between resulting groups after a fission event.

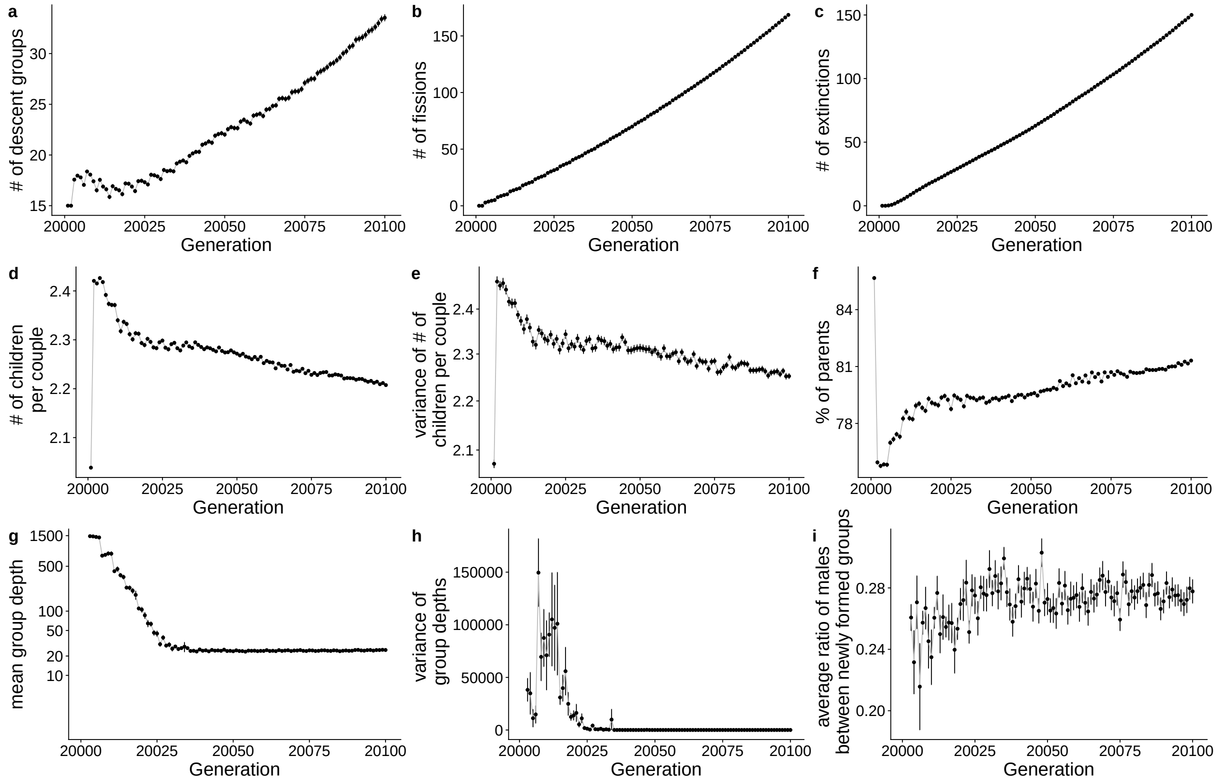

Supplementary Figure 25: Change in demographic metrics over time for simulations of the scenario 2h without post-fission migration or 2% intervillage migration

Means and their 95% confidence intervals calculated on all replicates are shown every two generations. **a**: average number of descent groups. **b**: average cumulated number of fissions. **c**: average cumulated number of extinctions. **d**: average number of children per couple (without taking into account single individuals). **e** average variance of number of children per couple (without taking into account single individuals). **f**: average percentage of female having children. **g**: average mean number of generations to the most recent common ancestors of descent groups. **h**: average variance of number of generations to the most recent common ancestors of descent groups. **i**: average ratio of number of individuals between resulting groups after a fission event.

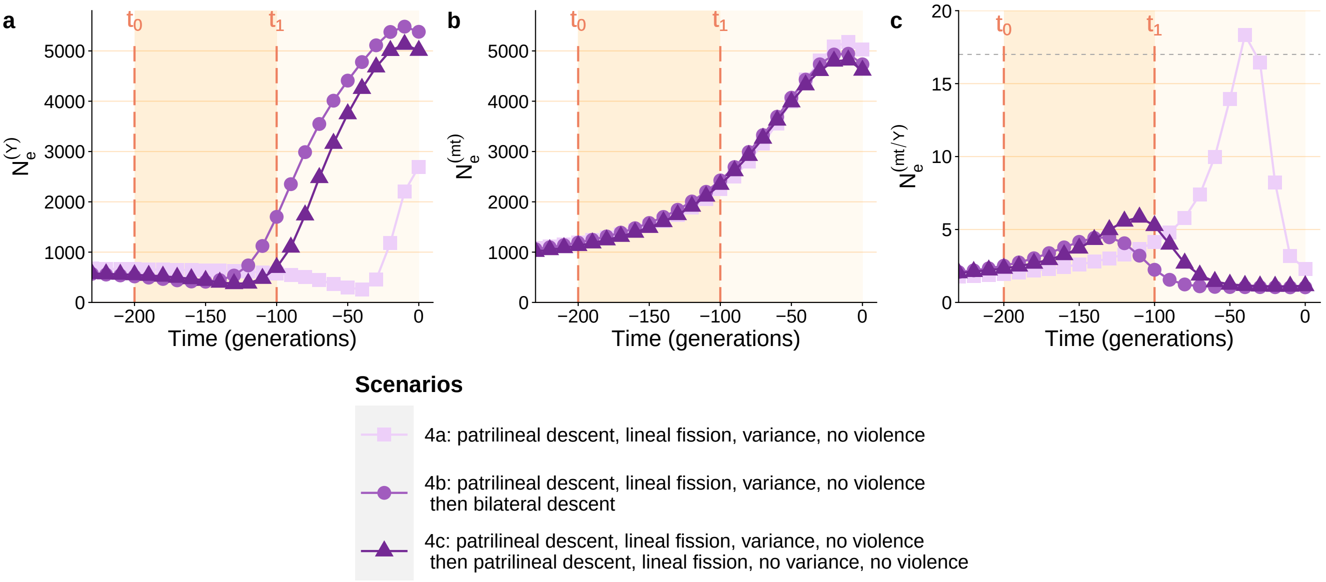

Supplementary Figure 26: Change in coalescent-based male and female effective population sizes over time under extended scenarios

In the scenario 4a, there is a transition at t_0, 200 generations before present, from panmixia to a patrilocal and patrilineal system with the settings of scenario 2g (i.e. lineal fission, variance in reproductive success between groups, and no violence). In the scenario 4b, there is a first transition at t_0 from panmixia to a patrilocal and patrilineal system with the settings of scenario 2g followed at t_1, 100 generations before present, by a transition to a bilateral system (with the settings of scenario 1). In the scenario 4c, there is a first transition at t_0 from panmixia to a patrilocal and patrilineal system with the settings of scenario 2g, followed by a transition at t_1 to a patrilocal and patrilineal system with the settings of scenario 2c (i.e. lineal fission, no variance in reproductive success between descent groups, no violence). See legend of Figure 2 for parameter values. Average Bayesian skyline plots of male effective population size (**a**), female effective population size (**b**), and female-to-male N_e ratio (**c**) over 200 replicates are shown. Values of means are provided in the Source Data file.

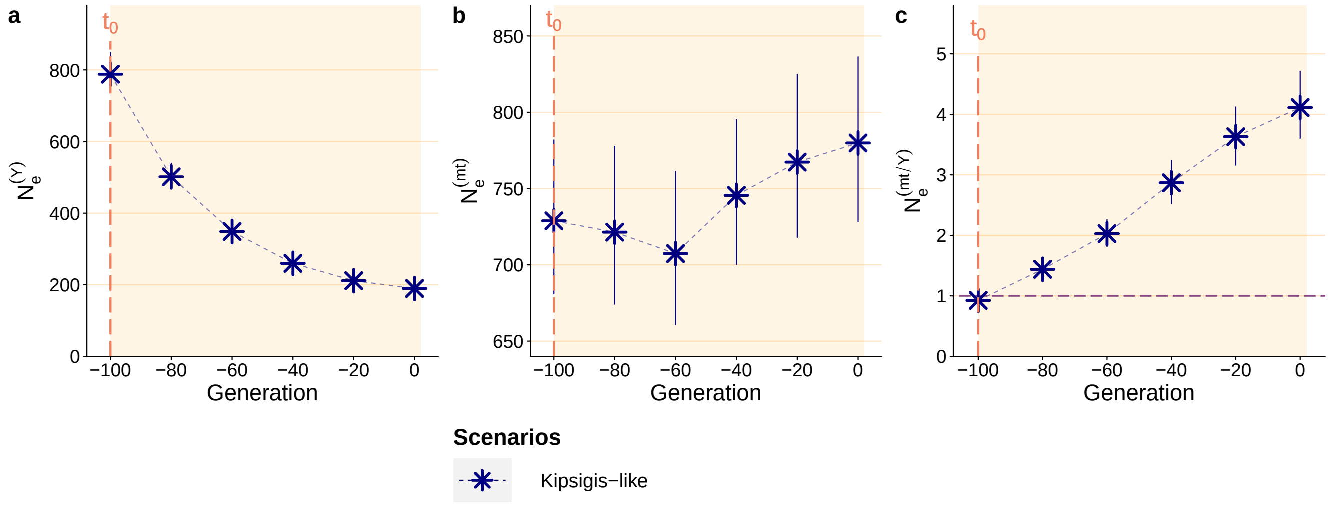

Supplementary Figure 27: Change in π-based male and female effective population sizes over time under the scenario with bilateral descent and polygyny

Patrilocal residence and polygyny are introduced at t_0, 100 generations before present, after a phase of panmixia. Male migration rate between villages is set to 0. π-based male effective population size (**a**), female effective population size (**b**) and female-to-male N_e ratios (**c**) averaged over 200 replicates are shown every 20 generations with the 95% confidence interval. Values of means and their 95% confidence intervals are provided in the Source Data file.

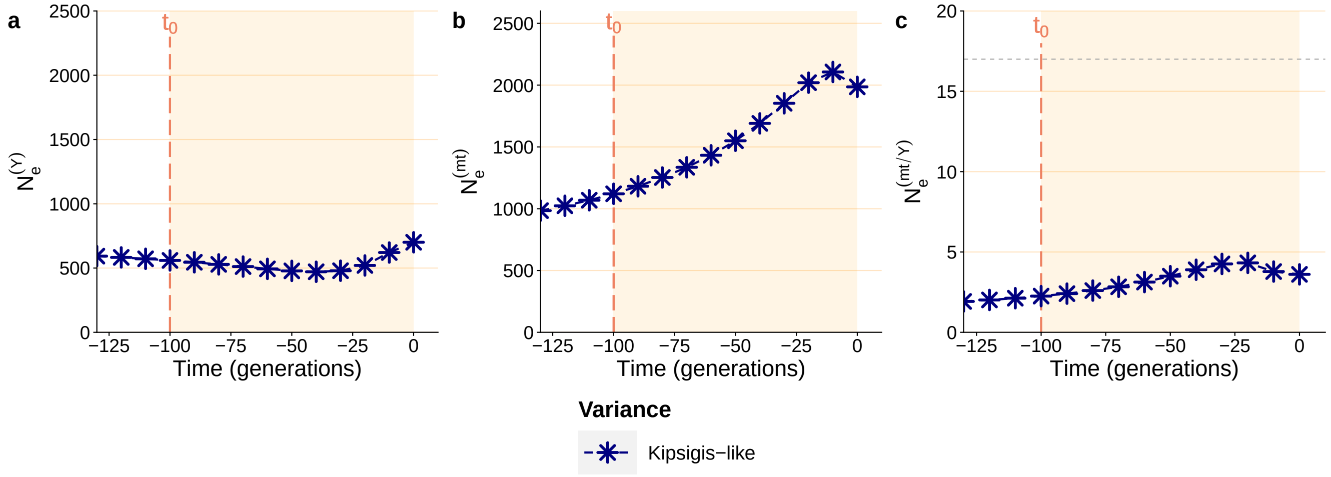

Supplementary Figure 28: Change in coalescent-based estimation of male and female effective population sizes over time under the scenario with bilateral descent and polygyny

Patrilocal residence and polygyny are introduced at t_0, 100 generations before present. Male migration rate between villages is set to 0. Average Bayesian skyline plots of male effective population size (**a**), female effective population size (**b**), and female-to-male N_e ratio (**c**) over 200 replicates are shown. Values of means are provided in the Source Data file.
