# Supplementary material for: Patrilineal segmentary systems provide a peaceful explanation for the post-Neolithic Y-chromosome bottleneck
Source: Nat Commun. 2024 Apr 24;15:3243. doi: 10.1038/s41467-024-47618-5 (PMC11043392; doi:10.1038/s41467-024-47618-5)
Supplement: Supplementary file 3 — Reporting Summary [file 41467_2024_47618_MOESM3_ESM.pdf]

nature portfolio

Corresponding author(s): Léa Guyon and Raphaëlle Chaix

Last updated by author(s): 27/03/2023

Reporting Summary

Nature Portfolio wishes to improve the reproducibility of the work that we publish. This form provides structure for consistency and transparency in reporting. For further information on Nature Portfolio policies, see our Editorial Policies and the Editorial Policy Checklist.

Statistics

For all statistical analyses, confirm that the following items are present in the figure legend, table legend, main text, or Methods section.

n/a	Confirmed	
☐	☒	The exact sample size (*n*) for each experimental group/condition, given as a discrete number and unit of measurement
☐	☒	A statement on whether measurements were taken from distinct samples or whether the same sample was measured repeatedly
☒	☐	The statistical test(s) used AND whether they are one- or two-sided<br>*Only common tests should be described solely by name; describe more complex techniques in the Methods section.*
☒	☐	A description of all covariates tested
☒	☐	A description of any assumptions or corrections, such as tests of normality and adjustment for multiple comparisons
☐	☒	A full description of the statistical parameters including central tendency (e.g. means) or other basic estimates (e.g. regression coefficient) AND variation (e.g. standard deviation) or associated estimates of uncertainty (e.g. confidence intervals)
☒	☐	For null hypothesis testing, the test statistic (e.g. *F*, *t*, *r*) with confidence intervals, effect sizes, degrees of freedom and *P* value noted<br>*Give P values as exact values whenever suitable.*
☐	☒	For Bayesian analysis, information on the choice of priors and Markov chain Monte Carlo settings
☒	☐	For hierarchical and complex designs, identification of the appropriate level for tests and full reporting of outcomes
☒	☐	Estimates of effect sizes (e.g. Cohen's *d*, Pearson's *r*), indicating how they were calculated

Our web collection on statistics for biologists contains articles on many of the points above.

Software and code

Policy information about availability of computer code

Data collection	Data was generated using:<br>1) a SLiM (v4.1) custom code simulating population socio-demographic scenarios and generating tree sequences (.trees files)<br>2) a Python (v3.8) custom code transforming the .trees files in VCF files for each chromosome, using tskit (v0.5.6), msprime (v1.3.0), pyslim (v1.0.4), numpy (v1.24.4), glob2 and argparse packages.<br>3) a Python custom code transforming the .trees files in nexus files, using tskit (v0.5.4), msprime (v1.2.0), pyslim (v1.0.1), numpy (v1.23.2), glob2 and argparse packages.<br><br>These scripts are available on github at: https://github.com/lea-guyon/PatriSim and on Zenodo at: https://zenodo.org/doi/10.5281/zenodo.10854123<br>Scripts can be run using the parameters listed in the file "scenarios_parameters.md". Packages requirements are available in the file "requirements.txt".
Data analysis	VCF files were analysed using:<br>1) custom Python scripts to compute summary statistics, using numpy (v1.14.4), glob2 and argparse packages.<br>2) a custom R script to generate figures, using ggplot2, cowplot and boot packages<br><br>nexus files are transformed using a Python script to be readable by the BEAUTi v1.10.5 software. Bayesian Skyline Plots are generated from these files using the software BEAST v1.10.5 and drawn using the script plot-skyline.R available on Art Poon's github.<br><br>The whole pipeline can be executed by running a single bash script.

The scripts are available on github at: https://github.com/lea-guyon/PatriSim and on Zenodo at: https://zenodo.org/doi/10.5281/zenodo.10854123
Scripts can be run using the parameters listed in the file "scenarios_parameters.md". Packages requirements are available in the file "requirements.txt".

For manuscripts utilizing custom algorithms or software that are central to the research but not yet described in published literature, software must be made available to editors and reviewers. We strongly encourage code deposition in a community repository (e.g. GitHub). See the Nature Portfolio guidelines for submitting code & software for further information.

Data

Policy information about availability of data

All manuscripts must include a data availability statement. This statement should provide the following information, where applicable:

- Accession codes, unique identifiers, or web links for publicly available datasets
- A description of any restrictions on data availability
- For clinical datasets or third party data, please ensure that the statement adheres to our policy

All data were generated by simulation and can be reproduced by running the scripts available on github at: https://github.com/lea-guyon/PatriSim. All means and their 95\% confidence intervals presented in Figures 2 to 6 and Supplementary Figures 3 to 17 and 26 to 28 were computed from simulated data and are provided in the Source Data file.

Research involving human participants, their data, or biological material

Policy information about studies with human participants or human data. See also policy information about sex, gender (identity/presentation), and sexual orientation and race, ethnicity and racism.

Reporting on sex and gender	N/A
Reporting on race, ethnicity, or other socially relevant groupings	N/A
Population characteristics	N/A
Recruitment	N/A
Ethics oversight	N/A

Note that full information on the approval of the study protocol must also be provided in the manuscript.

Field-specific reporting

Please select the one below that is the best fit for your research. If you are not sure, read the appropriate sections before making your selection.

☐ Life sciences ☐ Behavioural & social sciences ☒ Ecological, evolutionary & environmental sciences

For a reference copy of the document with all sections, see nature.com/documents/nr-reporting-summary-flat.pdf

Ecological, evolutionary & environmental sciences study design

All studies must disclose on these points even when the disclosure is negative.

Study description	We simulated different population socio-demographic scenarios in forward-time using SLiM 4. These scenarios include a transition from a panmictic population to a population structured in five patrilocal villages of size 300. The descent rule may be bilateral (individuals are affiliated with the kinship groups of both their parents) or patrilineal (individuals are affiliated with the kinship group of their father). When descent is patrilineal, villages are organised into several descent groups that may or may not experience varying levels of reproductive success, undergo random or lineal fission, and may or may not engage in violent competition with one another. For each scenario we ran 200 replicates and we measured the evolution of genetic diversity on uniparental markers (Y chromosome and mtDNA) over time. Male and female effective sizes were computed from:<br>1) the number of pairwise difference in the simulated population over time<br>2) Bayesian Skyline Plots (BSPs)
Research sample	To calculate the mean number of pairwise differences (pi), we sampled 100 individuals from the 5 simulated villages (20 per village) every 20 generations for each replicate of each scenario. In order to create the Bayesian Skyline Plots, we sampled 100 individuals from the 5 simulated villages (20 per village) at the end of the simulations for each replicate of each scenario. Descent group sizes, fission parameters, and variance in reproductive success between groups were set based on values reported in the literature for several segmentary patrilineal populations worldwide from 500 BP to the present, without mention of warfare between descent groups.
Sampling strategy	For the computation of pi, 20 individuals are sampled in each village (there are 5 villages in the simulated populations), 10 are males and 10 are females.

	For the BSPs, 20 individuals are sampled in each village, 10 males and 10 females.<br>In both cases, the sample sizes were chosen empirically to ensure that they represent the diversity of the village or population, and because they correspond to usual sample sizes in human population genetic studies.
Data collection	Output files are automatically written in a specific folder.
Timing and spatial scale	Simulated individuals are sampled from the end of the burn-in phase to the end of simulations every 20 generations.
Data exclusions	No data was excluded from the study.
Reproducibility	All scenarios were simulated for 200 replicates. Genetic diversity was measured for all replicates and averaged over all replicates. Similarly, Bayesian Skyline Plots were plotted for all replicates and averaged BSPs were computed.
Randomization	Not relevant since all data was simulated.
Blinding	No data collection phase.

Did the study involve field work? ☐ Yes ☒ No

Reporting for specific materials, systems and methods

We require information from authors about some types of materials, experimental systems and methods used in many studies. Here, indicate whether each material, system or method listed is relevant to your study. If you are not sure if a list item applies to your research, read the appropriate section before selecting a response.

Materials & experimental systems

n/a	Involved in the study
☒	☐ Antibodies
☒	☐ Eukaryotic cell lines
☒	☐ Palaeontology and archaeology
☒	☐ Animals and other organisms
☒	☐ Clinical data
☒	☐ Dual use research of concern
☒	☐ Plants

Methods

n/a	Involved in the study
☒	☐ ChIP-seq
☒	☐ Flow cytometry
☒	☐ MRI-based neuroimaging

Plants

Seed stocks	/
Novel plant genotypes	/
Authentication	/
